# Transferrin receptor 1 shedding by the pro-inflammatory iRhom–ADAM17 complex and ADAM10 regulates cellular iron uptake and ferroptosis

**DOI:** 10.1038/s12276-026-01731-1

**Published:** 2026-06-04

**Authors:** Katharina Schun, Cindy Rinkens, Daniel Mehling, Yan Yu, Sarah Knapp, Carolin Peschke, Friederike Sonnabend, Christine Lux, Alessa Pabst, Laura Charlier, Neele Schumacher, Aaron Babendreyer, Andreas Ludwig, Stefan Düsterhöft

**Affiliations:** 1 Institute of Molecular Pharmacology, Medical Faculty, RWTH Aachen University, Aachen, Germany; 2 GENEART AG/Thermo Fisher Scientific, Regensburg, Germany; 3 Institute of Biochemistry, Medical Faculty, Kiel University, Kiel, Germany; 4 Medical Faculty, Campus Düsseldorf/Krefeld, HMU Health and Medical University, Düsseldorf, Deutschland

**Keywords:** Diagnostic markers, Cell death, Molecular biology, Proteolysis

## Abstract

Iron homeostasis is a tightly regulated mechanism, wherein the uptake, transport, storage and export of iron are stringently controlled. Dysregulation and excessive iron uptake lead to iron-dependent programmed cell death called ferroptosis, a promising future cancer therapy target. Cellular iron uptake is limited by the surface presence of membrane-bound transferrin receptor 1 (TfR1). Soluble TfR1 is used as a major clinical marker to differentiate anemia types. Here we identify iRhoms, the regulatory interactors of the surface protease ADAM17, as substrate platforms. They bind TfR1 and facilitate ADAM17-mediated proteolytic TfR1 release (TfR1 shedding). Thereby, the iRhom–ADAM17 complex regulates TfR1 surface levels. Notably, TfR1 preferentially binds to pro-inflammatory iRhom2 over iRhom1, with the cytosolic N terminus of iRhom serving as a critical binding determinant. By CRISPR–Cas9-based knockout and pharmacological inhibition in vitro, in human primary endothelial cells as well as in ex vivo human lung slices, we also demonstrate that TfR1 is a shared substrate of ADAM10 and ADAM17. Functionally, we found that ADAM17-dependent TfR1 shedding reduces excessive iron uptake. By live cell imaging, we identified TfR1 shedding as a protective mechanism against ferroptosis. Moreover, reduced TfR1 shedding correlaśtes with elevated serum iron levels in ADAM17-hypomorphic mice, highlighting its systemic relevance for patho(physiological) iron homeostasis.

**Figure:**
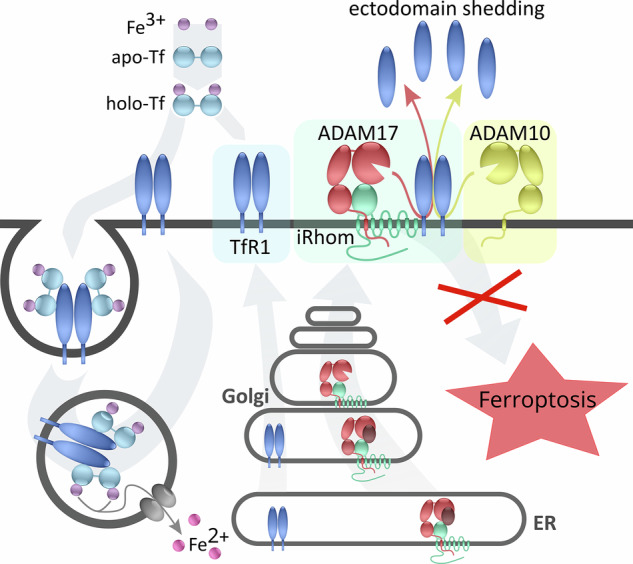
iRhom–ADAM17 complex and ADAM10-shed TfR1. Cellular iron uptake is facilitated by TfR1. TfR1 interacts with the substrate platform iRhom, which is a regulator of the protease ADAM17. The iRhom–ADAM17 complex and ADAM10 cleave TfR1, thereby releasing soluble TfR1. By this, iron overloaded is prevented. Therefore, ectodomain shedding of TfR1 is a protective mechanism to hinder ferroptosis.

iRhom–ADAM17 complex and ADAM10-shed TfR1. Cellular iron uptake is facilitated by TfR1. TfR1 interacts with the substrate platform iRhom, which is a regulator of the protease ADAM17. The iRhom–ADAM17 complex and ADAM10 cleave TfR1, thereby releasing soluble TfR1. By this, iron overloaded is prevented. Therefore, ectodomain shedding of TfR1 is a protective mechanism to hinder ferroptosis.

## Targeting TfR1 shedding offers new cancer therapy insights

Iron is a vital nutrient for all living organisms, crucial for processes such as oxygen transport and cellular functions. However, excess iron can lead to oxidative stress and ferroptosis, a form of cell death. This study explores the regulation of iron homeostasis, focusing on the shedding of transferrin receptor 1 (TfR1), a key player in iron uptake. Researchers identified iRhoms as platforms that bind TfR1, facilitating its shedding by the enzyme ADAM17. Furthermore an iRhom-independent shedding by the enzyme ADAM10 was identified. Using CRISPR–Cas9 knockout models and pharmacological inhibition, they demonstrated that inhibiting TfR1 shedding increases iron uptake, heightening sensitivity to ferroptosis. This finding is important, as it links TfR1 shedding to iron regulation and cell death, suggesting potential therapeutic targets for conditions such as cancer, where ferroptosis could be exploited. Future research could further elucidate the molecular mechanisms and explore therapeutic applications. This summary was initially drafted using artificial intelligence, then revised and fact-checked by the author.

## Introduction

Iron (Fe) is an essential nutrient required by all living organisms, playing a critical role in various biological processes throughout evolution. It is a key component of hemoglobin, and beyond its involvement in oxygen transport, iron also contributes to diverse metabolic processes and cellular functions such as mitochondrial respiration, immune function and DNA synthesis^1^.

While iron is essential for various biological processes, excessive iron accumulation can result in the generation of reactive oxygen species, thereby inducing oxidative stress. Such oxidative stress is harmful for the cell and can induce ferroptosis, a recently discovered form of iron-dependent programmed cell death^2^. Iron homeostasis and ferroptosis are critically linked to a variety of (patho)physiological processes such as infection control, autoimmunity, inflammation, aging, neurodegeneration and tumor suppression. Strikingly, ferroptosis-inducing drugs are already used in clinical trials for antitumor treatment^3^. Although ferroptosis is a key cellular process with crucial systemic relevance, the underlying regulatory mechanisms are still not completely understood.

**Fig. 1:**
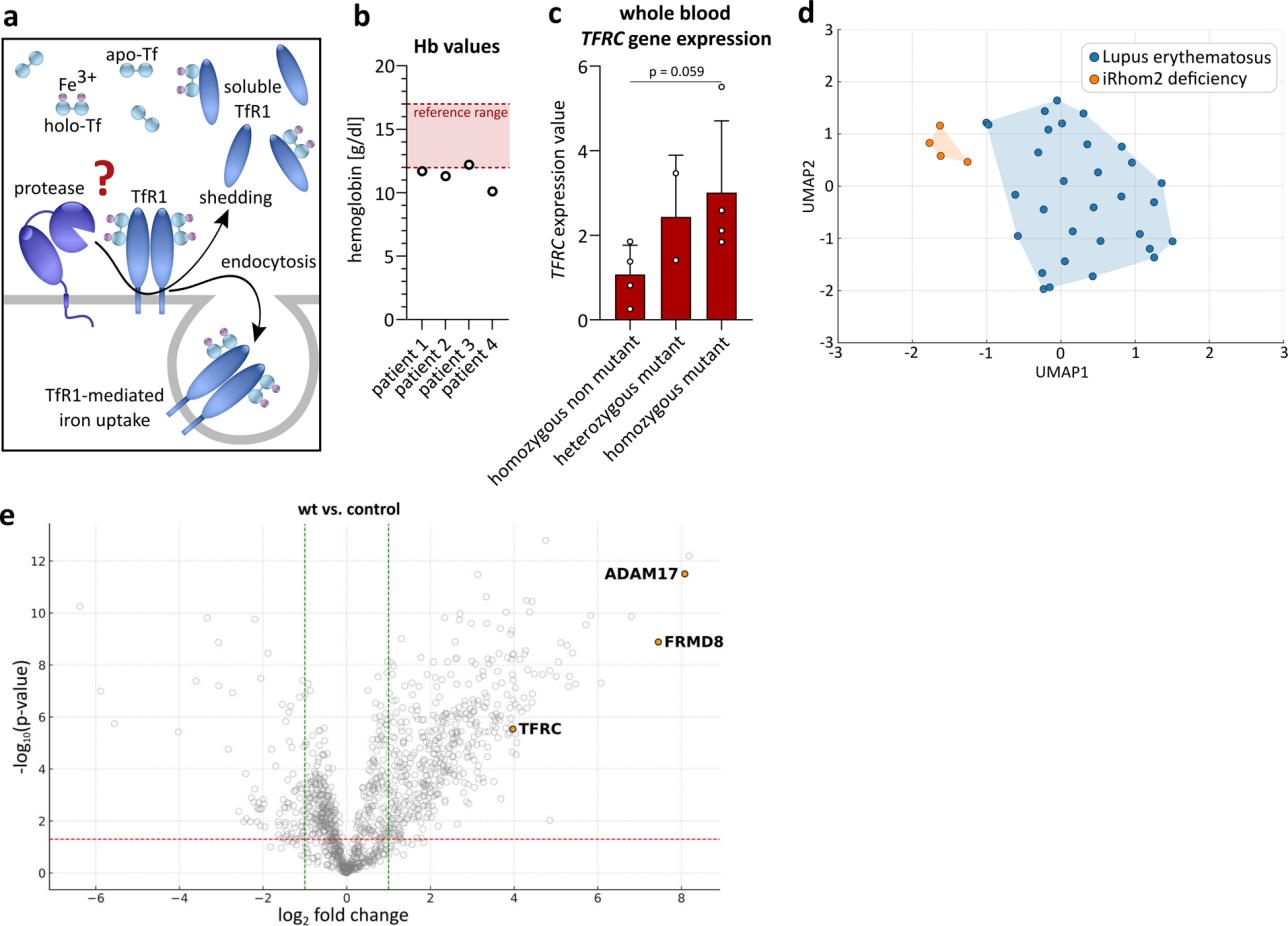
Iron metabolism is linked to iRhom2. **a**, Schematic overview of TfR1-mediated iron uptake in contrast to proteolytic generation of soluble TfR1 (shedding). Transmembrane metalloproteases have been implicated in the shedding of TfR1. **b**, Hemoglobin (Hb) values of four distinct patients with congenital iRhom2 deficiency were compared with the age-matched reference range^31^. **c**, The publicly available mRNA sequencing dataset from whole blood samples of patients with congenital iRhom2 deficiency (accession no. GSE184876) was reanalyzed for *TFRC* gene expression from four different homozygous nonmutant healthy controls, two different heterozygous mutant parents and four different homozygous mutant patients (*n* ≥ 2). Data are presented as mean + s.d. **d**, Whole blood mRNA sequencing data from patients with congenital iRhom2 deficiency^31^ (GSE184876) and those with chronic inflammatory lupus erythematosus^32^^,^^33^ (GSE112087) were reanalyzed. Gene expression profiles from these patient cohorts were compared with those of healthy controls. Genes associated with the following GO terms^35^^,^^36^ were compared for the selected datasets and visualized within a UMAP: GO:0006826 (iron ion transport), GO:0010039 (response to iron ion), GO:0006879 (intracellular iron ion homeostasis) and GO:0097707 (ferroptosis). Individual patient data points and the UMAP CHAs, which illustrate the distribution of both datasets, are shown. A more detailed gene-based comparison of reanalyzed mRNA sequencing data is shown in Supplementary Fig. 1. **e**, Volcano plot of interactome screen via mass spectrometry^25^, comparing control (GFP) and wt iRhom2. Significantly regulated proteins are marked by the red (*P* value of 0.05) and green (fold change of 2) borders. Known iRhom interactors, ADAM17 and FRMD8 as well as the novel iRhom2 interactor TfR1 (TFRC), are highlighted in orange.

Given that both iron overload and iron deficiency pose substantial health risks, iron homeostasis is tightly regulated to control its uptake, transport, storage and export^1,4^. The cellular iron uptake is largely mediated by transferrin (Tf) receptor 1 (TfR1)^5,6^. TfR1-dependent iron uptake is directly related to the surface expression of TfR1, which can be controlled in the long term by gene expression^4^. Rapid reduction of surface TfR1 can be achieved by cleavage of its ectodomain and subsequent release of soluble TfR1^7^, a general process that occurs with many surface proteins and is known as ectodomain shedding^8^. Importantly, circulating, solubilized TfR1, present in the bloodstream, serves as a critical marker for diagnosing iron deficiency. It is the main diagnostic marker to reliably differentiate between iron deficiency anemia and anemia of chronic disorders or anemia of chronic renal failure^9^. Moreover, it is a well-established diagnostic marker for erythropoietic activity^10^. Yet, the underlying molecular mechanisms of TfR1 shedding are poorly understood (Fig. 1a). Pharmacological data suggest that members of the metalloproteinase family can be implicated in TfR1 shedding^11^. However, the identity of the responsible sheddases is still lacking.

A disintegrin and metalloproteinase (ADAM)10 and ADAM17 are key shedding enzymes involved in many fundamental (patho)physiological processes, including development, differentiation, regeneration and inflammatory response^12–15^. This is due to their broad, in some cases overlapping, substrate spectrum, which includes membrane-bound forms of various cytokines (for example, tumor necrosis factor (TNF)), adhesion molecules (for example, L-selectin), growth factors (for example, epidermal growth factor (EGF) and transforming growth factor alpha (TGFα)) and receptors (for example, interleukin 6 receptor (IL6R))^16^. Hence, these ADAMs have to be tightly regulated. For ADAM17, the pseudoproteases iRhom1 and iRhom2 play a crucial role in its maturation and regulation^17–19^. Both iRhom1 and iRhom2 are catalytically inactive proteins belonging to the rhomboid superfamily of intramembrane proteases^20^. They form a complex with ADAM17 and promote its trafficking to the cell surface^21^. Recently, it has also been suggested that iRhoms serve not only as a transport and regulatory hub for ADAM17 but are also involved in ADAM17 substrate specificity, in addition to ADAM17’s direct substrate recognition functions^22,23^. However, a detailed understanding of the substrate recognition and specificity by the ADAM17-iRhom complex is still missing.

Here, we identify iRhoms as substrate platforms, which bind TfR1, leading to ADAM17-mediated shedding. We also define the cytosolic N-terminal region of iRhoms as a necessary TfR1 binding determinant. Furthermore, we identify TfR1 as a shared substrate of ADAM17 and ADAM10 through pharmacological inhibition in model cell lines, primary endothelial cells and human precision-cut lung slices, as well as CRISPR–Cas9-mediated knockout (KO) in model cell lines and analyses of serum from transgenic ADAM17-hypomorphic mice. Finally, we show that inhibition of TfR1 shedding increases TfR1 cell surface expression, causing elevated Tf and iron uptake, which then promotes higher sensitivity to ferroptosis.

## Methods

### Cell culture

HEK293 cells (from the American Type Culture Collection, CRL-1573, RRID: CVCL-0045) and iRhom2 KO, iRhom1/iRhom2 double KO (dKO) as well as ADAM17 and/or ADAM10 KO cells^24^ were cultured in a humidified incubator at 37 °C with 5% CO_2_ in DMEM 10%, unless otherwise stated. DMEM 10% consists of DMEM high-glucose (Sigma-Aldrich) supplemented with 10% fetal bovine serum (FBS) (PanBiotech), 60 mg/l penicillin (Sigma-Aldrich) and 100 mg/l streptomycin (Sigma-Aldrich). Cell culture plates were coated with 40 µg/ml collagen G (Sigma-Aldrich) in PBS (Sigma-Aldrich), before culturing iRhom2 KO or iRhom1/iRhom2 dKO HEK293 cells. Stable cell lines were produced as described before^25^.

Human umbilical vein endothelial cells (HUVECs) were isolated as described before^26^ from umbilical cords of cesarean sections provided by Aachen University Hospital in accordance with ethics vote EK241/18. The cells were cultured in endothelial cell growth medium MV2 with supplement pack (PromoCell), 60 mg/l penicillin (Sigma-Aldrich) and 100 mg/l streptomycin (Sigma-Aldrich) in a humidified incubator at 37 °C with 5% CO_2_.

### Generation of iRhom2 KO and iRhom1/iRhom2 dKO HEK293 cells

iRhom2 KO and iRhom1/iRhom2 dKO HEK293 cells were generated using a CRISPR–Cas9-based KO system as described in the Supplementary Methods.

### Cloning

A plasmid containing the sequence of human TfR1 (mKOet-TFR-20, Plasmid #57935, Addgene) was used as a template, and a 3xmyc tag was fused by overlapping PCR. To generate different iRhom variants, side-directed mutagenesis was performed by using overlapping PCR. According to the instructions, the NEBuilder HiFi DNA assembly master mix (NEB) was used to insert different variants into a vector. To fuse the iRhom variants to a 3xHA tag, a pMOWS backbone was used. Respectively, a plasmid with zeocin resistance (pMOWS_zeo) or a puromycin resistance (pMOWS_puro) was used.

### Immunoprecipitation

Precipitation of proteins from supernatant or cell lysate was performed as described in the Supplementary Methods by using magnetic beads.

### Western blotting

The samples were separated using SDS–polyacrylamide gel electrophoresis and subsequently transferred onto polyvinylidene difluoride membranes (0.45 µm pore size; Millipore, Immobilon-FL). Following this, the membranes were blocked with a blocking solution of 5% (w/v) nonfat dry milk in Tris-buffered saline (TBS; 50 mM Tris, 150 mM NaCl, pH 7.4) for 20 min at room temperature. The membranes were then incubated overnight at 4 °C with primary antibodies diluted in 0.1% Tween-TBS and 1% (w/v) BSA. The primary antibodies used in this study, along with their respective dilutions, are enumerated as follows: mouse anti-HA (1:2000, 901502, Biolegend), goat anti-HA (1:1000, TA150086, Thermo Fisher), rabbit anti-TfR1 (1:1000, ab84036, Abcam), mouse anti-TfR1 (1:2000, 13-6800, Thermo Fisher), rabbit anti-ADAM17 (1:1666, ab39162, Abcam), mouse anti-myc (1:2500, ab39, Abcam), rabbit anti-ADAM10 (1:2000, AB19026, Merck/Sigma-Aldrich), mouse anti-iRhom2 (1:1000, MAB10048, R&D systems), mouse anti-GAPDH (1:2000, MA5-15738, Thermo Fisher), rabbit anti-alkaline phosphatase (1:1000, ab124772, Abcam), mouse anti-flotillin-1 (1:1000, 610821, BD Bioscience), rabbit anti-myc coupled with horseradish peroxidase (HRP; 1:2000, ab19312, Abcam) and rabbit anti-HA-HRP (1:3333, HAM0601, R&D Systems). Following three washing steps with 0.1% Tween-TBS, the membranes were then incubated with a secondary antibody for 1.5 h at room temperature. These secondary antibodies were used: donkey anti-rabbit Alexa Fluor Plus 800 (1:100,000, A32808, Thermo Fisher), donkey anti-goat DyLight 488 (1:100,000, SA5-10086, Thermo Fisher), donkey anti-mouse IRDye 680RD (1:100,000, ab216778, Abcam), goat anti-mouse DyLight 680 (1:100,000, 35519, Thermo Fisher), goat anti-rabbit-HRP (1:100,000, 111-036-003, ImmunoResearch Laboratories) and goat anti-mouse-HRP (1:100,000, 115-036-003, ImmunoResearch Laboratories). All secondary antibodies were diluted 1:100,000 in 0.1% Tween-TBS and 1% (w/v) BSA. Additional washing steps were performed, including one wash with 0.1% Tween-TBS and two washes with TBS. The stained proteins were subsequently detected via fluorescence using the ChemiDoc MP Imaging System (Bio-Rad) or the Odyssey 9120 imager system (LI-COR). Chemiluminescence reaction was used to detect weak signals using Amersham ECL Prime solutions (Cytiva) or SuperSignal West Femto Maximum Sensitivity Substrate (Thermo Fisher) with the ChemiDoc MP Imaging System (Bio-Rad). Quantification was performed by measuring band intensities using Image Studio Lite software v5.2 (LI-COR). For replicated experiments, band intensities of each sample were referred to the sum of all samples of the western blot (sum of the replicates)^27^.

### qPCR

RNA was extracted using the RNeasy Kit (Qiagen) and quantified photometrically with the NanoDrop (Peqlab). Reverse transcription of 500 ng RNA was performed using the PrimeScript RT Reagent Kit (Takara Bio Europe). Following the manufacturer’s protocol, PCR reactions were conducted with the iTaq Universal SYBR Green Supermix (Bio-Rad). The quantitative PCR (qPCR) protocol was run on the CFX Connect Real-Time PCR Detection System (Bio-Rad) with the following settings: initial denaturation at 95 °C for 5 min, followed by 45 cycles of denaturation at 95 °C for 10 s, annealing for 30 s and amplification at 72 °C for 15 s. To quantify *hTFRC* expression, the forward primer CTTCAATCACACTCAGTTTCCACCA and the reverse primer CAGTCTCCTTCCATATTCCCAAACAG were used with an annealing temperature of 59 °C. Expression levels were normalized to the mRNA levels of the human reference genes *hRPL13A* (forward primer: GCCCTACGACAAGAAAAGCG; reverse primer: TACTTCCAGCCAACCTCGTGA; annealing temperature: 60 °C) and *hCYC1* (forward primer: AGCTATCCGTGGTCTCACC; reverse primer: CCGCATGAACATCTCCCCATC; annealing temperature: 59 °C). To determine the efficiency of each qPCR run, the mean efficiency of all samples meeting specific criteria were calculated using linear regression with LinReg software (v2018.0, Dr. J.M. Ruitjer, Academic Medical Centre, Amsterdam). Exclusion criteria are enumerated as follows: absence of amplification, absence of a plateau phase, noisy samples and PCR efficiency deviating more than 5% from the group median. CFX Maestro software (v1.1, Bio-Rad) was used for relative quantification.

### Serum from mice

In this study, serum of ADAM17^wt/wt^ and ADAM17^ex/ex^ mice (V 242 - 55995/2019(26-3/19)), provided by the Institute of Biochemistry, Kiel University, was used for TfR1 enzyme-linked immunosorbent assay (ELISA) and determination of serum iron levels.

### Human precision-cut lung slices

Supernatant of human lung slices generated in a previous study^28^ were used for analyzing TfR1 shedding via ELISA.

### ELISA

Supernatants from treated HEK239 cells or HUVECs were collected and cleared from cell debris by centrifugation (16,000*g*, 5 min, 4 °C). Human precision-cut lung slice samples were generated in a previous study^28^. Released soluble TfR1 was measured by a human TfR1 ELISA (DY2474, R&D Systems) according to the manufacturer’s instructions. For chromogenic reaction, the BM Blue POD substrate (Roche) was used. Serum levels of soluble TfR1 of ADAM17^wt/wt^ and ADAM17^ex/ex^ mice were measured by murine TfR1 ELISA (ab243674, Abcam), which was performed according to the manufacturer’s instructions. The optical density was determined by SpectraMax iD3 (Molecular Devices).

### Tf uptake assay

HEK293 cells were starved with DMEM without FBS for 2 h at 37 °C. Afterward, the cells were stored on ice for 10 min and washed with cold Live Cell Imaging Solution (Thermo Fisher) with 20 nM glucose and 1% (w/v) BSA (LCIS +/+). Cells were incubated with 25 µg/ml Alexa Fluor 488 conjugated Tf (Thermo Fisher) in LCIS +/+ for 20 min at 37 °C. The endocytosis was stopped on ice and samples were washed with cold LCIS +/+. The cells were collected with accutase (PanBiotech), fixed with 2% paraformaldehyde and used for flow cytometry analysis.

### Flow cytometry

Staining was performed using PBS supplemented with 0.2% (w/v) BSA as the assay buffer, with all steps conducted at 4 °C or on ice. Cells (2 × 10^5^) were incubated with the primary antibody mouse anti-TfR1 (1:500, ab1086, Abcam), mouse anti-HA (1:500, 901502, Biolegend), mouse anti-ADAM17 (1:50, MAB 9301, R&D systems) or mouse anti-ADAM10 (1:500, MAB1427, R&D systems) for 1 h. Following incubation, the cells were washed three times with 400 μl assay buffer. The secondary antibody allophycocyanin-conjugated anti-mouse (1:200, 115-135-164, Jackson ImmunoResearch) was added, and the cells were incubated in the dark for 45 min. Tf uptake assay samples were not stained with antibodies. After washing twice with assay buffer, cells were resuspended in PBS. The fluorescence signal was evaluated using flow cytometry (LSR-Fortessa, BD Biosciences), and data were analyzed with FlowJo V10 software. The median fluorescence intensity (MFI) was determined to assess cell surface localization. For replicated experiments, the MFI of each sample was normalized to the sum of all samples (sum of the replicates)^27^.

### Ferroptosis assay by live cell imaging

HEK293 cells were seeded in a 12-well plate. At a confluence of around 30%, cells were starved with DMEM without FBS for 2 h at 37 °C. After washing the cells with PBS, they were treated with 10 µM RSL3 (HY-100218A, MedChemExpress) and 250 nM Incucyte Cytotox Dye Red (Sartorius) in DMEM with 20% FBS. Treated cells were cultured in a humidified incubator at 37 °C with 5% CO_2_ for 24 h. Pictures were taken every 30 min using Incucyte SX5 and analyzed via Incycyte software v2024A (Sartorius).

### Iron levels

Murine serum iron levels of ADAM17^wt/wt^ and ADAM17^ex/ex^ mice were quantified according to the manufacturer´s protocol using VITROS Chemistry Products Fe Slides (1515808), VITROS Chemistry Products Calibrator Kit 4 (1204668) and the VITROS Chemistry System by the Institute of Laboratory Animal Science, University Hospital Aachen.

### Reanalysis of mRNA sequencing data

The publicly available mRNA sequencing datasets were retrieved from the Gene Expression Omnibus (GEO) database^29,30^. Differential gene expression analysis was initially performed using GEO2R (https://www.ncbi.nlm.nih.gov/geo/geo2r/)^30^, an online tool that uses the R packages limma and GEOquery. Local R software with the following packages was used for further analysis: tidyverse (2.0.0), data.table (1.17.0), AnnotationDbi (1.68.0), org.Hs.eg.db (3.20.0), Deseq2 (1.46.0) and uwot (0.2.3). Experimental groups were defined according to the original study to identify genes with significant differential expression. mRNA sequencing data derived from whole blood samples of patients with congenital iRhom2 deficiency^31^ (GSE184876), patients with chronic inflammatory lupus erythematosus^32,33^ (GSE112087) and peripheral blood mononuclear cell samples from a patient harboring a point mutation in TfR1 (TfR1^R22W^ (^34^); GSE243237) were reanalyzed. Gene expression profiles from these patient cohorts were compared with those of healthy controls, focusing on genes associated with the following Gene Ontology (GO) terms^35,36^: GO:0006826 (iron ion transport), GO:0010039 (response to iron ion), GO:0006879 (intracellular iron ion homeostasis) and GO:0097707 (ferroptosis). In addition, the expression of the following genes was examined: *IL6, CXCL8 (IL8), TNF, ADAM10, ADAM17, RHBDF1* and *RHBDF2*. Furthermore, a Uniform Manifold Approximation and Projection (UMAP) was calculated using R with the fold change of each individual patient relative to average healthy control samples. Custom Python scripts (Python 3.11.9) with Seaborn (0.13.2), Matplotlib (3.8.2) and SciPy (1.12.0) were used to visualize data distributions and to perform convex hull area (CHA) calculation to represent the spatial distribution of the data.

### Statistics

Statistical analysis was performed using the generalized linear mixed model analysis (PROC GLIMMIX, SAS 9.4, SAS Institute). Data were analyzed for the optimal distribution, using Akaike information criterion and the Bayesian information criterion. The residual analysis and the Kolmogorov–Smirnov test were also used as diagnostics. In the case of heteroscedasticity (according to the covtest statement), the degrees of freedom were adjusted using the Kenward–Roger approximation. All *P* values were adjusted for multiple comparisons by the false discovery rate.

## Results

### Iron metabolism is dysregulated in iRhom2-deficient patients

Loss-of-function mutations in *RHBDF2* (encoding iRhom2) lead to dysregulation of the immune system with chronic susceptibility to infections^31^. We reanalyzed whole blood mRNA sequencing data from these patients compared with healthy controls and found dysregulation of iron-related genes (Supplementary Fig. 1). Moreover, patients with congenital iRhom2 deficiency showed decreased hemoglobin (Hb) values (Fig. 1b). Notably, *TFRC* (encoding TfR1) gene expression was increased in healthy people heterozygous for the mutation and even more increased in homozygous mutant patients, compared with the homozygous nonmutant healthy people (Fig. 1c). Remarkably, genes involved in iron metabolism and iron homeostasis were distinctly regulated between the iRhom2-deficient patients and patients with chronic inflammatory lupus erythematosus^37^, whom have also been shown to have elevated systemic inflammatory markers such as IL6 and elevated TfR1 expression (Fig. 1d and Supplementary Fig. 1). This indicates that the dysregulation of iron metabolism-related genes may not be solely the result of the inflammation due to infection in the iRhom2-deficient patients but may be the result of a regulatory role of iRhom2. Indeed, in our recent iRhom2 interactome screen, we identified TfR1 as a significant hit and, hence, a putative interactor of iRhoms^25^, indicating a direct connection between iRhoms and iron metabolism (Fig. 1e).

### TfR1 interacts with iRhom2 on the cell surface

**Fig. 2:**
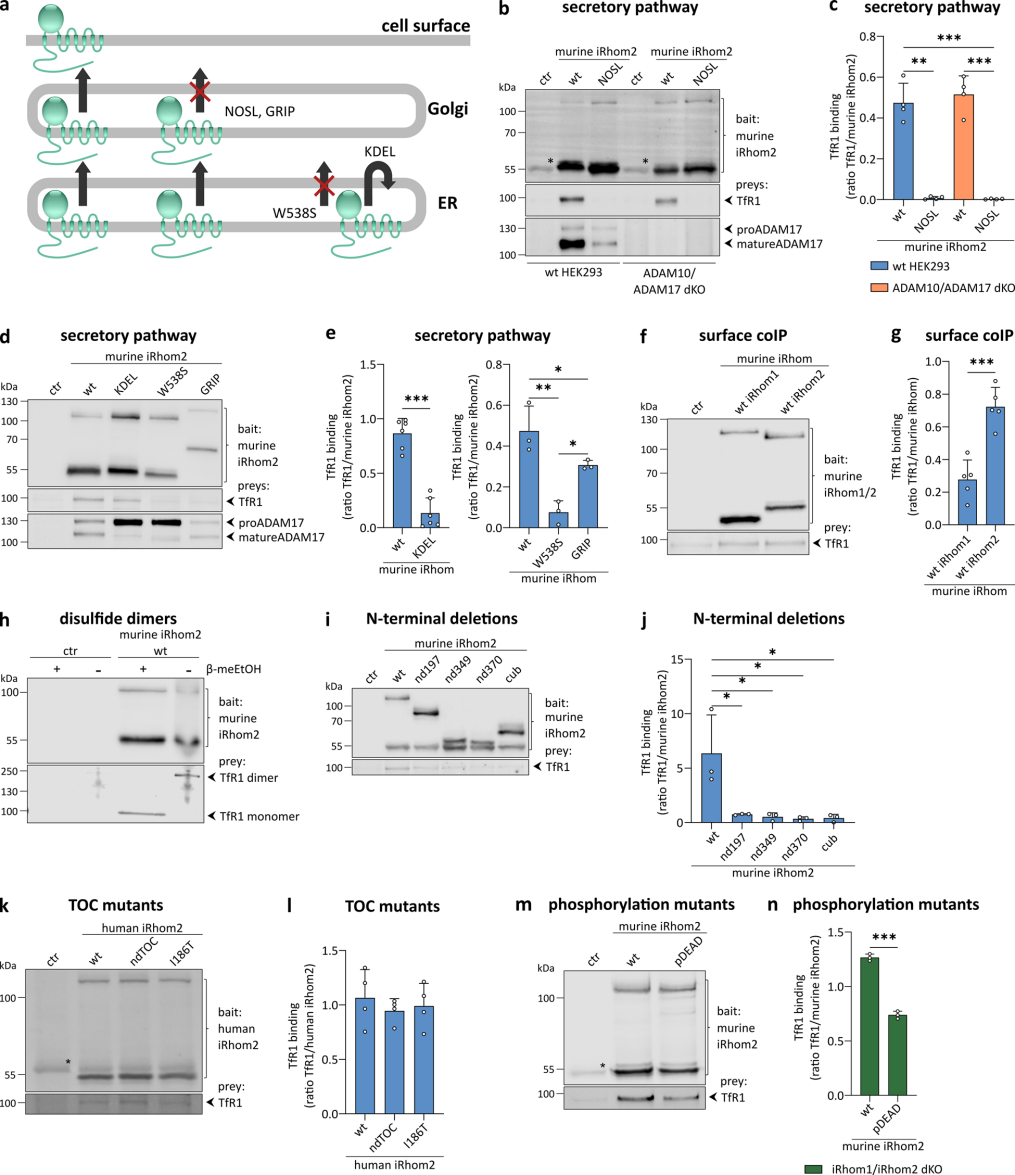
TfR1 interacts with iRhom2 at the cell surface, with the cytosolic N terminus of iRhom2 serving as a determinant of this binding. **a**, Schematic representation of the iRhom2 variants used in this study. Shown are different iRhom2 mutations that trap iRhom2 at differing stages in the secretory pathway. The murine iRhom2_W538S does not exit the ER, while murine iRhom2_KDEL (added C-terminal signal sequence KDEL) is sorted back to the ER^25^. By contrast, murine iRhom2_NOSL (S644N, P691A and A692S) and murine iRhom2_GRIP (added C-terminal GRIP domain) do not leave the Golgi apparatus. All other variants used in this study successfully reach the cell surface (wt murine iRhom1, wt murine iRhom2, murine iRhom2_pDEAD^46^, murine iRhom2_nd197, murine iRhom2_nd349, murine iRhom2_nd370, murine iRhom2_cub, wt human iRhom1, wt human iRhom2, human iRhom2_ndTOC and human iRhom2_I186T^41^). In the murine iRhom2_pDEAD variant, all potential phosphorylation sites in the iRhom N terminus were mutated to alanine. The murine iRhom2_nd197 variant lacks amino acids 1–197, while the murine iRhom2_cub variant lacks amino acids 1–268. The two other deletions extend beyond the region deleted in the cub variant, with murine iRhom2_nd349 lacking amino acids 1–349 and murine iRhom2_nd370 lacking nearly the entire N terminus (amino acids 1–370). The N-terminal deletion variant of the human iRhom2 spanning the TOC mutation sides (human iRhom2_ndTOC) only removes a small region of the N terminus (Δ182–196). **b**–**n**, The HEK293 cells used in this study were either transduced with the indicated iRhom variants or GFP (ctr) as a negative control. If not stated otherwise, wt HEK293 cells were used. In (**b**), additionally, ADAM10/ADAM17 dKO cells (ADAM10/ADAM17 dKO) were used (*n* = 4), and in (**m**), iRhom1/iRhom2 dKO cells (iRhom1/iRhom2 dKO) were used (*n* = 3). CoIPs were performed to analyze the binding between TfR1 and iRhom variants. The iRhom variants with an HA tag were used as bait, and the TfR1 binding was quantified by calculating the levels of coprecipitated TfR1 (prey) relative to the precipitated iRhom variants (bait) (ratio bound TfR1/iRhom). To analyze TfR1–iRhom binding at the cell surface, a surface coIP was performed (Methods) (*n* = 5) (**f**). The samples were prepared with reducing (+β-mercaptoethanol (β-meEtOH)) or nonreducing (−β-meEtOH) conditions to compare the TfR1 monomer or TfR1 dimer binding to wt murine iRhom2 (*n* = 3) (**h**). The corresponding lysate controls of all coIPs are shown in Supplementary Fig. 2. The bands marked with an asterisk indicate a fragment of the antibody used for immunoprecipitation. The higher iRhom band represents the full-length iRhom, and the lower iRhom band represents a cleaved fragment of iRhom^40^. *n* = 4 (**c**), *n* ≥ 3 (**d** and **e**), *n* = 5 (**g**), *n* = 3 (**i** and **j**), *n* = 4 (**k** and **l**), *n* = 3 (**n**). Data are presented as mean + s.d. from at least three independent experiments. Significant differences were indicated by asterisks (*P < 0.05, **P < 0.01, ***P < 0.001). **b**, Representative western blot of coIP of wt HEK239 and ADAM10/ADAM17 KO cells with control (GFP), wt murine iRhom2, murine iRhom2_NOSL. **c**, Quantification of (**b**). **d**, Representative western blot of coIP of wt HEK239 cells with control (GFP), wt murine iRhom2, murine iRhom2_KDEL, murine iRhom2_W538S, murine iRhom2_GRIP. **e**, Quantification of (**d**). **f**, representative western blot of surface coIP of wt HEK239 cells with control (GFP), wt murine iRhom1, murine iRhom2. **g**, Quantification of (**f**). **h**, Representative western blot of coIP of wt HEK239 cells with control (GFP), wt murine iRhom2. **i**, Representative western blot of coIP of wt HEK239 cells with control (GFP), wt murine iRhom2, murine iRhom2_nd197, murine iRhom2_nd349, murine iRhom2_nd370, murine iRhom2_cub. **j**, Quantification of (**i**). **k**, Representative western blot of coIP of wt HEK239 cells with control (GFP), wt murine iRhom2, murine iRhom2_ndTOC, murine iRhom2_I186T. **l**, Quantification of (**k**). **m**, Representative western blot of coIP of iRhom1/iRhom2 dKO HEK293 cells with control (GFP), wt murine iRhom2, murine iRhom2_pDEAD. **n**, Quantification of (**m**).

To validate the interactome data, we conducted in vitro experiments to verify the possible molecular interaction between iRhom2 and TfR1. iRhom2 is the main interactor of ADAM17 already in its zymogen form (proADAM17) in the endoplasmic reticulum (ER). It is also essential for transporting ADAM17 to the Golgi for maturation and reaching the cell surface^38,39^. Indeed, co-immunoprecipitations (coIPs) of HA-tagged wild type (wt) murine iRhom2 in HEK293 cells showed the binding of both ADAM17 forms (Fig. 2b,d and Supplementary Fig. 2a,f). Importantly, also endogenous TfR1 was coprecipitated with iRhom2 (Fig. 2b,d). In addition, we were able to coprecipitate iRhom2, using endogenous TfR1 as bait (Supplementary Fig. 2d,e). To analyze where iRhom2 meets TfR1 in the cell, we developed a set of different iRhom2 variants, which are limited to different cell compartments through the secretory pathway (Fig. 2a). In contrast to wt murine iRhom2, the ability of the murine iRhom2_NOSL (S644N, P691A, A692S; murine iRhom2 with no surface localization) and murine iRhom2_GRIP (iRhom2 with C-terminal GRIP domain) variants to reach the cell surface is drastically reduced (Supplementary Fig. 2b,c,i,j). Yet, both still coprecipitated proADAM17 and mature ADAM17 (Fig. 2b,d and Supplementary Fig. 2a,f), indicating that they still can reach the Golgi. By contrast, the binding of these Golgi-limited variants to TfR1 was significantly reduced (Fig. 2b–e). We additionally used the previously described ER-resident iRhom2 variants murine iRhom2_KDEL and murine iRhom2_W538S^25^. CoIPs of wt murine iRhom2, murine iRhom2_NOSL and murine iRhom2_GRIP contained pro and mature ADAM17, while coIPs of the ER-trapped iRhom2 variants precipitated mostly proADAM17 (Fig. 2d and Supplementary Fig. 2f–h). This is in line with the known steps of the iRhom-depended ADAM17 maturation, on its way to the cell surface^16^. Murine iRhom2_KDEL and murine iRhom2_W538S, similar to murine iRhom2_NOSL and murine iRhom2_GRIP, also coprecipitated significantly less TfR1 compared with the wt murine iRhom2. The ER-trapped variants and murine iRhom2_NOSL also precipitated less TfR1 compared with the Golgi-trapped murine iRhom2_GRIP (Fig. 2c,e). This indicates that the interaction mainly takes place after the main stages of the secretory pathway when TfR1 and iRhom2 are being transported to the cell surface. On this basis, we carried out surface coIPs. Indeed, endogenous TfR1 was coprecipitated via iRhom2 from the cell surface of HEK293 cells expressing HA-tagged wt murine iRhom2 (Fig. 2f and Supplementary Fig. 2k). This shows that the iRhom2–TfR1 interaction persists on the cell surface. Whereas iRhom2 is the inactive rhomboid predominantly expressed in immune cells, iRhom1 is present in most cells^16^. Therefore, we also used HEK293 cells expressing wt murine iRhom1 for the surface precipitation. TfR1 was also coprecipitated from the plasma membrane with wt murine iRhom1, albeit to a significantly lesser extent than with wt murine iRhom2 (Fig. 2g).

To investigate whether iRhom2 and TfR1 only interact indirectly via ADAM17, HEK293 cells with ADAM10 and ADAM17 dKO (ADAM10/ADAM17 dKO) were used. TfR1 could be coprecipitated via wt murine iRhom2 but again not via murine iRhom2_NOSL (Fig. 2b,c). This shows that the interaction of iRhom2 and TfR1 does not depend on the presence of ADAM17. As TfR1 is present on the plasma membrane as a disulfide bond-connected homodimer, we used nonreducing sample preparation conditions to demonstrate that indeed the functional TfR1 dimer interacts with iRhom2 (Fig. 2h and Supplementary Fig. 2l).

In summary, we have shown that TfR1 does interact in its dimeric form with iRhom2 and that this interaction mainly occurs in the last stages of the secretory pathway and at the cell surface.

### The cytosolic N terminus of iRhom2 is an important determinant for TfR1 binding

iRhom1 and iRhom2 are largely homologous; however, their most substantial differences are located in the N terminus^40^. As we had observed differential binding of TfR1 to iRhom1 and iRhom2 at the cell surface, the influence of the iRhom2 N terminus on TfR1–iRhom2 complex formation was examined further. To this end, various N-terminal deletion variants (murine iRhom2_nd197, _nd349 and _nd370)^41^ were overexpressed in HEK293 cells. In addition, the iRhom2 cub variant (murine iRhom2_cub), which occurs naturally in mice, was included. This partial deletion of the N terminus has been associated with a curly hair phenotype in affected mice^42–44^. Notably, all murine N-terminal deletion variants exhibited significantly reduced TfR1 coprecipitation, highlighting the importance of the iRhom2 N terminus for iRhom2–TfR1 complex formation (Fig. 2i,j and Supplementary Fig. 2m). Single point mutations also exist in the N terminus of human iRhom2. These mutations are associated with tylosis with esophageal cancer (TOC) in humans and are involved in 14-3-3 binding^41,45^. iRhom2 mutations disrupting this site showed no difference in TfR1 precipitation compared with the wt human iRhom2 (Fig. 2k,l and Supplementary Fig. 2n).

The N terminus of iRhom contains several phosphorylation sites important for ADAM17 regulation and 14-3-3 binding^46,47^. To investigate whether these sites are relevant for TfR1 binding to iRhoms, HEK293 cells with an iRhom1 and iRhom2 dKO were produced and validated (Supplementary Fig. 2p–r and Supplementary Fig. 5b). These KO cells were reconstituted with wt murine iRhom2 or an iRhom2 variant in which phosphorylation sites were mutated (murine iRhom2_pDEAD^46^). The results demonstrated that TfR1 interacts significantly less with the iRhom2_pDEAD variant, suggesting that complex formation is phosphorylation dependent (Fig. 2m,n and Supplementary Fig. 2o).

Taken together, these results show a complex formation between iRhom2 and TfR1, which is dependent on the N terminus of iRhom and possibly on its phosphorylation status.

### TfR1 is shed by iRhom–ADAM17 complex and ADAM10

**Fig. 3:**
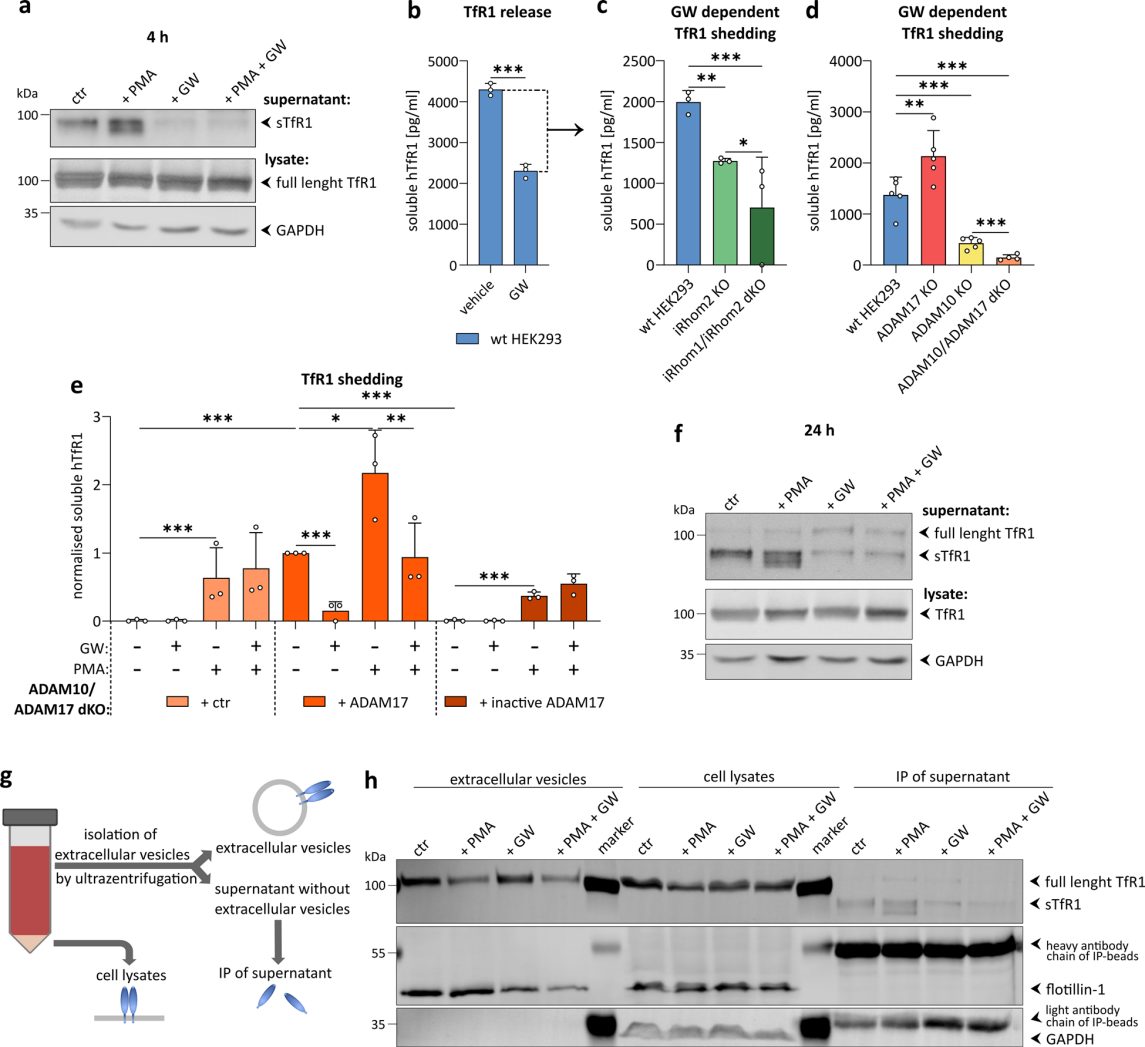
TfR1 is shed by iRhom–ADAM17 complex and ADAM10. **a**, HEK293 cells (transduced with HA-tagged wt murine iRhom2 and myc-tagged TfR1) were treated for 4 h with 100 nM PMA (PKC-dependent ADAM17 activator) and/or 10 µM GW (ADAM10 and ADAM17 inhibitor). Dimethylsulfoxide (DMSO) was used as a vehicle control (ctr). The supernatant was then used for immunoprecipitation (IP) of soluble TfR1 (sTfR1), while the lysates were used to detect noncleaved TfR1 (*n* = 1). GAPDH was used as a loading control. **b**–**e**, ELISA was used to determine the amount of soluble, endogenous TfR1 (**b** and **e**). Therefore, wt HEK293 cells (**b**), iRhom2 KO and iRhom1/iRhom2 dKO cells (*n* = 3) (**c**) or ADAM17 KO, ADAM10 KO and ADAM10/ADAM17 dKO cells (*n* ≥ 4) (**d**) were used. Cell were incubated for 24 h with 3 µM GW (ADAM10 and ADAM17 inhibitor). DMSO was used as a vehicle control. The supernatant was collected after 24 h. ADAM10- and ADAM17-independent shedding was subtracted from the total unstimulated shedding, ensuring that only ADAM10- and ADAM17-dependent shedding (GW dependent TfR1 shedding) is shown in (**c**) and (**d**). Raw data are shown in Supplementary Fig. 3a,b. ADAM10/ADAM17 dKO cells were transiently transfected with ADAM17, inactive ADAM17 or GFP (ctr) as a control (**d**). **e**, Cells were treated for 24 h with 100 nM PMA and/or 3 µM GW. DMSO was used as a vehicle control (−/−). Levels of soluble hTfR1 were measured by ELISA and normalized to ADAM10/ADAM17 dKO cells + ADAM17 treated with DMSO as a vehicle control (*n* = 3). Non-normalized data are shown in Supplementary Fig. 3c. Data are presented as mean + s.d. from at least three independent experiments. Significant differences are indicated by asterisks (*P < 0.05, **P < 0.01, ***P < 0.001). **f**, HEK293 cells (transduced with HA-tagged wt murine iRhom2 and myc-tagged TfR1) were treated for 24 h with 100 nM PMA (PKC-dependent ADAM17 activator) and/or 10 µM GW (ADAM10 and ADAM17 inhibitor). DMSO was used as a vehicle control (ctr). The supernatant was then used for IP of TfR1, while the lysates were used to detect noncleaved TfR1 (*n* = 1). GAPDH was used as a loading control. **g**, To isolate extracellular vesicles from the supernatant, the supernatant was separated from cells by centrifugation. The cell pellet was used to generate cell lysates. The extracellular vesicles were isolated from the supernatant via ultracentrifugation. Afterward, the remaining supernatant was used for IP of soluble TfR1 (sTfR1). **h**, Cells were treated as described in **f**, and samples were processed as described in **g**. Extracellular vesicles, cell lysates and IP of supernatant samples were used for western blot analysis. GAPDH was used as a loading control for cell lysates. The presence of flotillin-1, while GAPDH was absent, was used as a control for purified extracellular vesicles. Different antibody bands (heavy and light antibody chain) attributable to the used IP beads were detected in the western blot analysis of the IP samples. (*n* = 4).

As TfR1 can be cleaved by proteases and iRhom functions as a regulatory interactor of the sheddase ADAM17, we sought to determine whether ADAM17 is capable of shedding TfR1. To investigate this, HEK293 cells overexpressing myc-tagged TfR1 were used to precipitate soluble TfR1 from the supernatant. We indeed were able to detect soluble TfR1 in unstimulated cells (Fig. 3a). Following a 4-h stimulation with the phorbol ester phorbol 12-myristate 13-acetate (PMA), a protein kinase C (PKC)-dependent ADAM17 activator, an increased amount of soluble TfR1 was detected in the supernatant, accompanied by the appearance of a second band in the western blot (Fig. 3a). Furthermore, inhibition with GW280264X (GW), an ADAM10 and ADAM17 inhibitor, resulted in reduced soluble TfR1 in unstimulated and PMA-stimulated cells (Fig. 3a).

To further dissect the TfR1 shedding, soluble TfR1 release was determined in various HEK293 KO cell lines. We found that only a fraction of released TfR1 was GW-sensitive (Fig. 3b and Supplementary Fig. 3a). The GW-insensitive fraction may originate from TfR1 associated with extracellular vesicles, as previously reported^48^, or from cleavage by a GW-insensitive sheddase. In the following analyses, we focused on GW-sensitive TfR1 shedding. Wt HEK293 cells exhibited constitutive TfR1 shedding, which was decreased in HEK293 iR2 KO and even more in HEK293 iR1/iR2 dKO cells (Fig. 3c). This reduction demonstrates the iRhom dependency of ADAM17-mediated TfR1 shedding. However, iRhom deficiency could not completely abolish TfR1 release. Notably, unstimulated TfR1 shedding was significantly increased in ADAM17 KO cells compared with wt HEK293 cells (Fig. 3d and Supplementary Fig. 3b). This suggests that, in the absence of ADAM17, TfR1 shedding may be mediated by ADAM10, which is in line with a compensatory upregulation of ADAM10 surface levels in ADAM17 KO cells (Supplementary Fig. 5h,i). TfR1 shedding was significantly reduced in ADAM10 KO cells, indicating ADAM10-dependent TfR1 shedding. Nevertheless, ADAM10 KO cells still exhibited higher TfR1 shedding compared with ADAM10/ADAM17 dKO cells, confirming that ADAM17 also contributes to unstimulated TfR1 shedding (Fig. 3d and Supplementary Fig. 3b), which is also in line with the iRhom dependency of TfR1 shedding.

As ADAM10 typically mediates constitutive shedding, whereas ADAM17 is often activated under stimulatory conditions, TfR1 shedding was also analyzed after PMA stimulation. ADAM10/ADAM17 dKO HEK293 cells were rescued with expression of wt ADAM17, which resulted in constitutive ADAM17-mediated TfR1 shedding that could be inhibited by GW (Fig. 3e and Supplementary Fig. 3c). Moreover, PMA stimulation enhanced TfR1 shedding, and this effect was suppressed by GW, demonstrating PMA-induced ADAM17-dependent shedding (Fig. 3e). As an additional control, cells were transfected with an inactive ADAM17 variant^49^, which yielded results comparable to those observed in ADAM10/ADAM17 dKO cells transfected with the control vector (Fig. 3e). Interestingly, cells expressing control or inactive ADAM17 treated with PMA also showed an increase in soluble TfR1, which however was not sensitive to GW inhibition (Fig. 3e). This release may be attributed to the release of extracellular vesicles, as TfR1 is known to be also released via extracellular vesicles and PMA has been reported to induce extracellular vesicle secretion^50^.

In addition to a soluble cleaved TfR1, we detected full-length TfR1 in the supernatant of HEK293 cells TfR1 after 24 h (Fig. 3f). To investigate whether the full-length TfR1 precipitated from the supernatant was attributed to extracellular vesicles, ultracentrifugation was used (Fig. 3g,h). Indeed, we found full-length TfR1 was released via extracellular vesicles, as described earlier^48^ (Fig. 3h).

In summary, these results demonstrate that both the iRhom–ADAM17 complex as well as ADAM10 are principle sheddases for TfR1.

### TfR1 interacts preferentially with iRhom2 compared with iRhom1

**Fig. 4:**
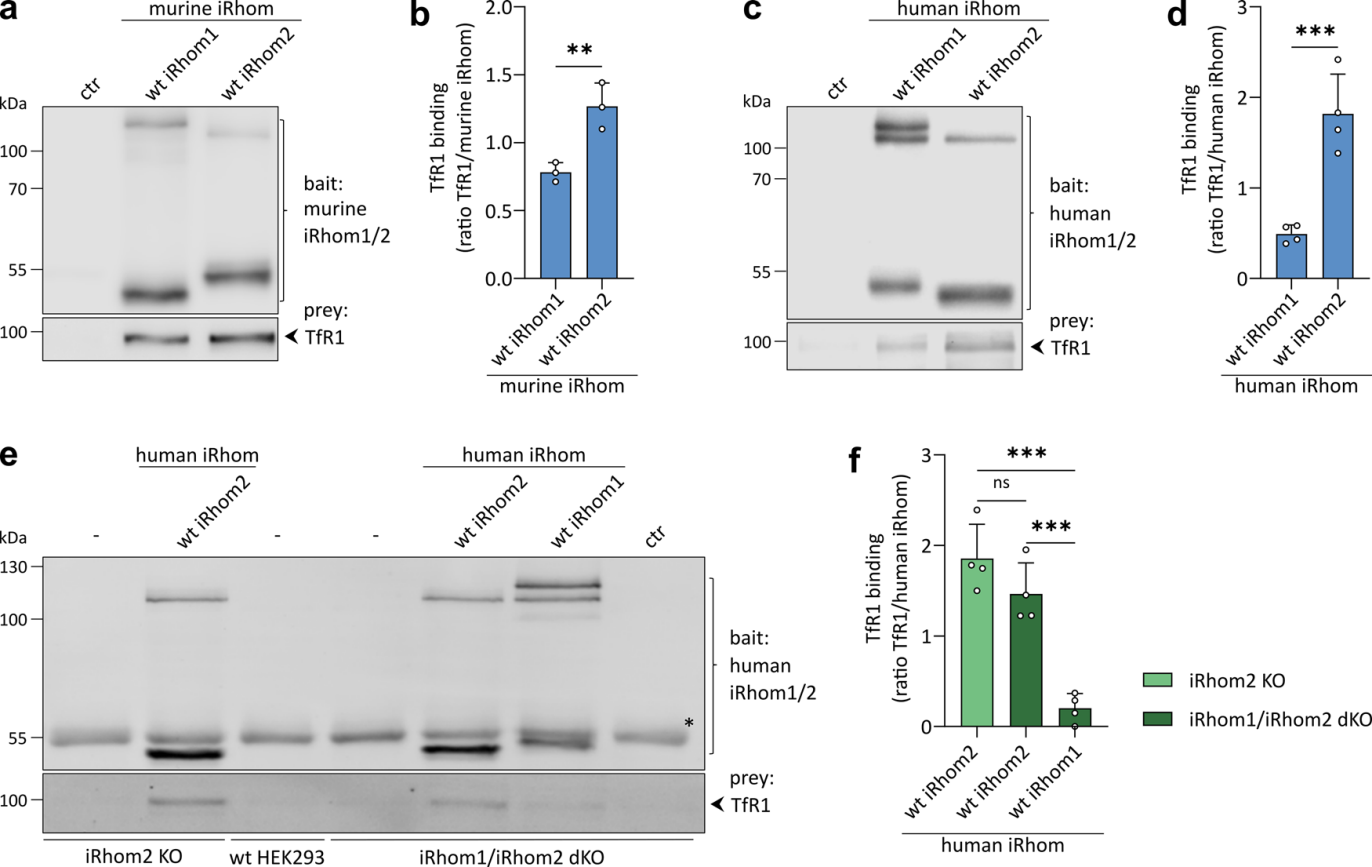
TfR1 interacts preferentially with iRhom2 compared with iRhom1. **a**–**f**, HEK293 cells stably expressing the indicated iRhom variants or GFP (ctr) as a negative control were used. iR2 KO cells and iR1/iR2 dKO cells were used in *n* = 3 (**a** and **b**) and *n* = 4 (**c** and **d**) wt HEK293 cells and in *n* = 4 wt HEK293 cells (**e** and **f**). CoIPs were performed to analyze the binding between TfR1 and iRhom variants. The iRhom variants with HA tag were used as bait, and the TfR1 binding was quantified by calculating the levels of coprecipitated TfR1 (prey) relative to the precipitated iRhom variants (bait) (**b**, **d** and **f**). Corresponding lysate controls are shown in the Supplementary Fig. 4. The band marked with an asterisk indicates a fragment of the antibody used for immunoprecipitation. The higher iRhom band represents the full-length iRhom and the lower iRhom band represents a cleaved fragment of iRhom^40^. Data are presented as mean + s.d. from at least three independent experiments. Significant differences are indicated by asterisks (*P < 0.05, **P < 0.01, ***P < 0.001).

It has previously been suggested that iRhom1 and iRhom2 are involved in substrate recognition and substrate selectivity, in addition to ADAM17’s direct substrate recognition functions^22,23^. Indeed, we were able to show that murine iRhom2 interacts with the ADAM17 substrates TGFα and amphiregulin (AREG), but not the ADAM10 substrate betacellulin (BTC), in an ADAM17-independent manner (Supplementary Fig. 4a,b). As our surface coIP already suggested that iRhom1 may bind TfR1 with lower affinity than iRhom2, we conducted a more detailed investigation to determine whether the binding of the newly identified ADAM17 substrate, TfR1, differs between iRhom1 and iRhom2. HEK293 cells were transduced with murine or human iRhom1 and iRhom2 and subsequently used for coIPs. TfR1 was found to coprecipitate with all iRhom variants. However, for both the murine and human iRhoms, iRhom1 exhibited lower TfR1 binding compared with iRhom2 (Fig. 4a–d and Supplementary Fig. 4c,d). To further validate these findings, HEK293 iRhom2 KO and HEK293 iRhom1/iRhom2 dKO cells (Supplementary Fig. 2p–r and Supplementary Fig. 5b) were reconstituted with either human iRhom1 or human iRhom2, each carrying an HA tag. This approach ensured that the observed effects were not influenced by endogenously expressed iRhoms. Once again, we demonstrated that iRhom1 binds TfR1 to a lesser extent than iRhom2 (Fig. 4e,f and Supplementary Fig. 4e).

These results indicate that iRhoms function as a platform for ADAM17 substrates including TfR1. Moreover, we identified that iRhom2 serves as an important hub for TfR1 but that iRhom1 is still able to bind TfR1. This is consistent with our other results showing that the KO of both iRhoms has a stronger effect than the KO of iRhom2 alone (Fig. 3b).

### ADAM10/ADAM17-mediated TfR1 shedding impacts TfR1 protein levels and Tf uptake

**Fig. 5:**
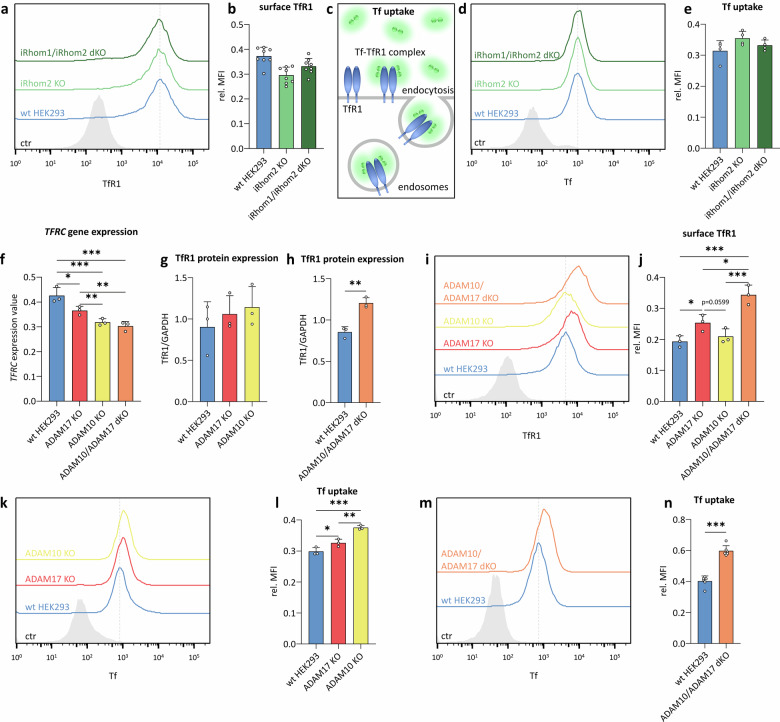
ADAM10/ADAM17-mediated TfR1 shedding impacts TfR1 protein levels and Tf uptake. **a**,**b**, The surface levels of endogenous TfR1 were measured via flow cytometry using wt HEK293 cells, iRhom2 KO and iRhom1/iRhom2 dKO cells (*n* = 8). (a = representative histogramm; b = quantification) **c**, A schematic overview of the Tf uptake assay. Fluorescent Tf is taken up by the cells via endocytosis of the Tf–TfR1 complex. **d**,**e**,**k**–**n**, Tf uptake of wt HEK293, iRhom2 KO, iRhom1/iRhom2 dKO (**d** (representative histogramm) and **e** (quantification) (*n* = 4), ADAM17 KO, ADAM10 KO (**k** (representative histogramm) and **l** (quantification)) (*n* = 3) and ADAM10/ADAM17 dKO (**m** (representative histogramm) and **n** (quantification)) (*n* = 6) cells was quantified by flow cytometry. **f**–**j**, *TFRC* gene expression (**f**) (*n* = 3), TfR1 protein levels (**g** (wt HEK293, ADAM17 KO, ADAM10 KO cells) and **h** (wt HEK293, ADAM10/ADAM17 dKO cells)) (*n* = 3) and TfR1 surface levels (**i** (representative histogramm) and **j** (quantification)) (*n* = 3) were determined for wt HEK293, ADAM17 KO, ADAM10 KO and ADAM10/ADAM17 dKO cells. TfR1 protein levels were quantified relative to the loading control GAPDH. The corresponding western blots are shown in Supplementary Fig. 5. Data are presented as mean + s.d. from at least three independent experiments. Significant differences are indicated by asterisks (*P < 0.05, **P < 0.01, ***P < 0.001).

The surface levels of TfR1 are essential for its main function, which is the uptake of iron-loaded Tf. Hence, a Tf uptake assay was performed. Fluorescent Tf was incubated with cells and its uptake via the endocytosis of TfR1 was measured by fluorescence intensity analysis via flow cytometry (Fig. 5c). The ability to take up Tf was not changed in iRhom2 KO or iRhom1/iRhom2 dKO cells (Fig. 5d,e). This is in line with no or only minimal changes in TfR1 surface levels, total protein levels or gene expression levels in iRhom-deficient HEK293 cells (Fig. 5a,b and Supplementary Fig. 5a–c).

Interestingly, *TFRC* gene expression was reduced with ADAM17 KO, ADAM10 KO and ADAM10/ADAM17 dKO (Fig. 5f). By contrast, there was no significant difference in TfR1 total protein levels for ADAM17 KO or ADAM10 KO but a significant increase in total TfR1 protein levels in ADAM10/ADAM17 dKO cells (Fig. 5g,h and Supplementary Fig. 5d–i). Remarkably, TfR1 surface levels were not changed for ADAM10 KO but were increased for ADAM17 KO and even more increased in ADAM10/ADAM17 dKO cells (Fig. 5i,j and Supplementary Fig. 5f–i). However, both ADAM17 KO and ADAM10 KO cells take up significantly more fluorescent Tf, compared with wt HEK293 cells (Fig. 5k,l). Notably, ADAM10/ADAM17 dKO cells took up much more Tf than wt HEK293 cells, ADAM17 KO or ADAM10 KO cells (Fig. 5m,n).

Taken together, while TfR1 levels and Tf uptake appear to be compensated in iRhom KO cells, ADAM10/ADAM17 KOs have a profound effect on TfR1 total protein levels, TfR1 surface levels and, importantly, Tf uptake, which cannot be compensated by downregulation of *TFRC* gene expression.

### ADAM10 or ADAM17 KO increase susceptibility to RSL3-inducible ferroptosis

**Fig. 6:**
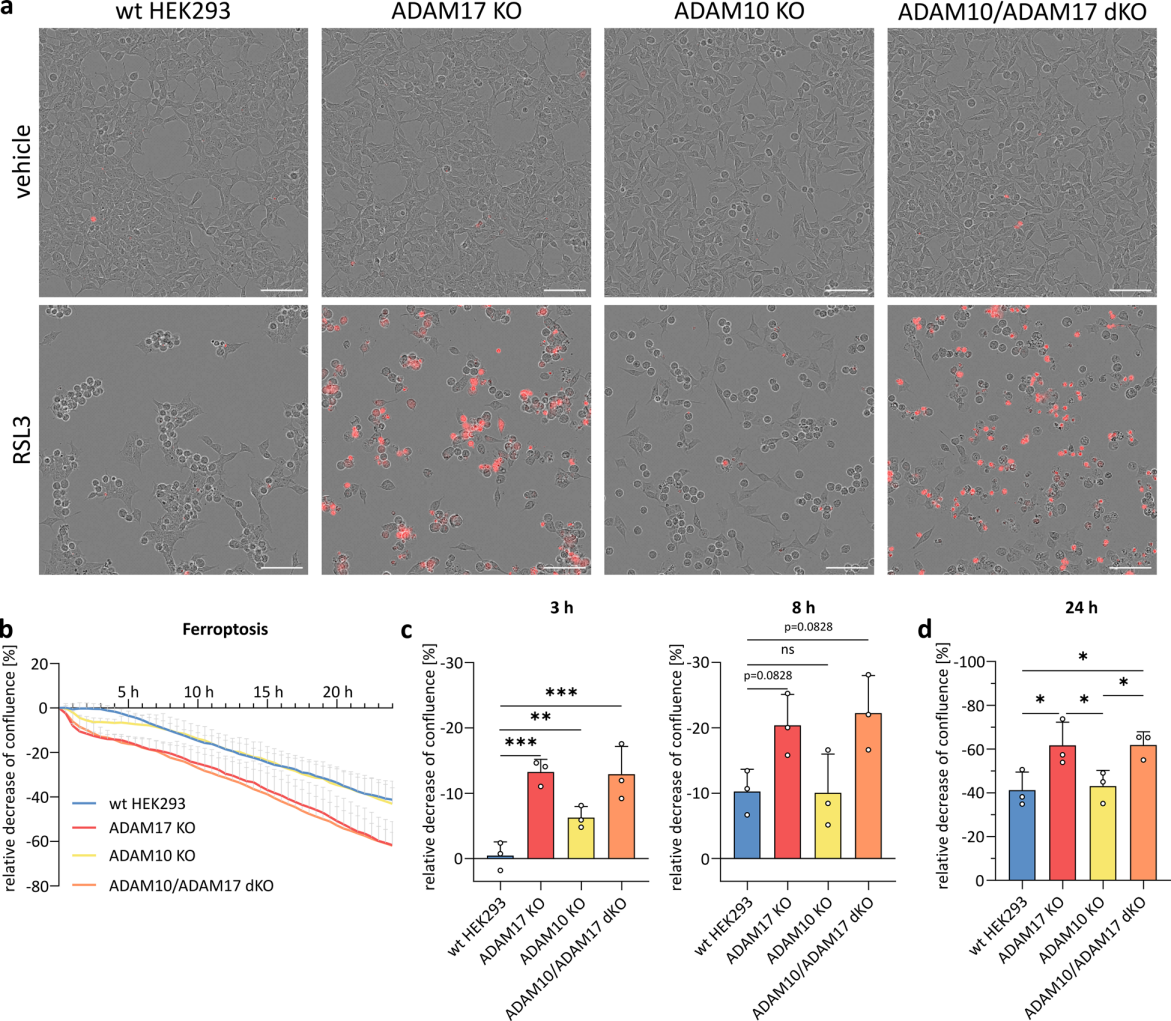
ADAM10 or ADAM17 KO increase susceptibility to RSL3-induced ferroptosis. Ferroptosis was induced by treating wt HEK293, ADAM17 KO, ADAM10 KO or ADAM10/ADAM17 dKO HEK 293 cells with 10 µM RSL3 for 24 h. 250 nM Incucyte Cytotox Dye Red was used to identify adherent dead cells (red staining). **a**, Representative pictures of HEK293 cells after 24 h RSL3 treatment (scale bar, 100 µm). DMSO was used as a vehicle control. **b**–**d**, The relative percental decrease of confluence in RSL3-treated cells was normalized to the cell growth of vehicle-treated cells over 24 h (**b**), after 3 h and 8 h (**c**) and after 24 h (**d**) (*n* = 3). Corresponding data are shown in Supplementary Fig. 6. Data are presented as mean + s.d. from at least three independent experiments. Significant differences are indicated by asterisks (*P < 0.05, **P < 0.01, ***P < 0.001).

Increased iron uptake into the cell can lead to iron overload, which can result in the highly regulated iron-dependent cell death, known as ferroptosis^2,51^. As increased Tf uptake was observed in ADAM10 and/or ADAM17 KO cells, which would lead to higher intracellular iron levels, the susceptibility of these cells to ferroptosis was investigated. HEK293 cells with ADAM10 and/or ADAM17 KO were treated with the ferroptosis inducer RSL3 for 24 h. Cell viability was assessed by live cell imaging in combination with cell death staining (Fig. 6a). In this setup, short- and long-term effects can be distinguished in the different KOs. Up to 3 h, wt HEK293 cells did not respond to RSL3 treatment, but in comparison, a significantly higher number of KO cells died (Fig. 6c). For ADAM17 KO cells, this difference remained after 8 h and 24 h, while this was not observed for ADAM10 KO cells at these time points. Whereas long-term RSL3 treatment only inhibited cell growth in wt HEK293 cells and HEK293 ADAM10 KO cells, it resulted in increased cell death in HEK293 ADAM17 KO and HEK293 ADAM10/ADAM17 dKO cells (Fig. 6b–d and Supplementary Fig. 6a,b).

Overall, the results show that ADAM17- and, to a lesser extent, ADAM10-dependent TfR1 shedding is important for preventing excess iron uptake and ferroptosis.

### Release of TfR1 ex vivo and in vivo depends on ADAM17/ADAM10 activity

**Fig. 7:**
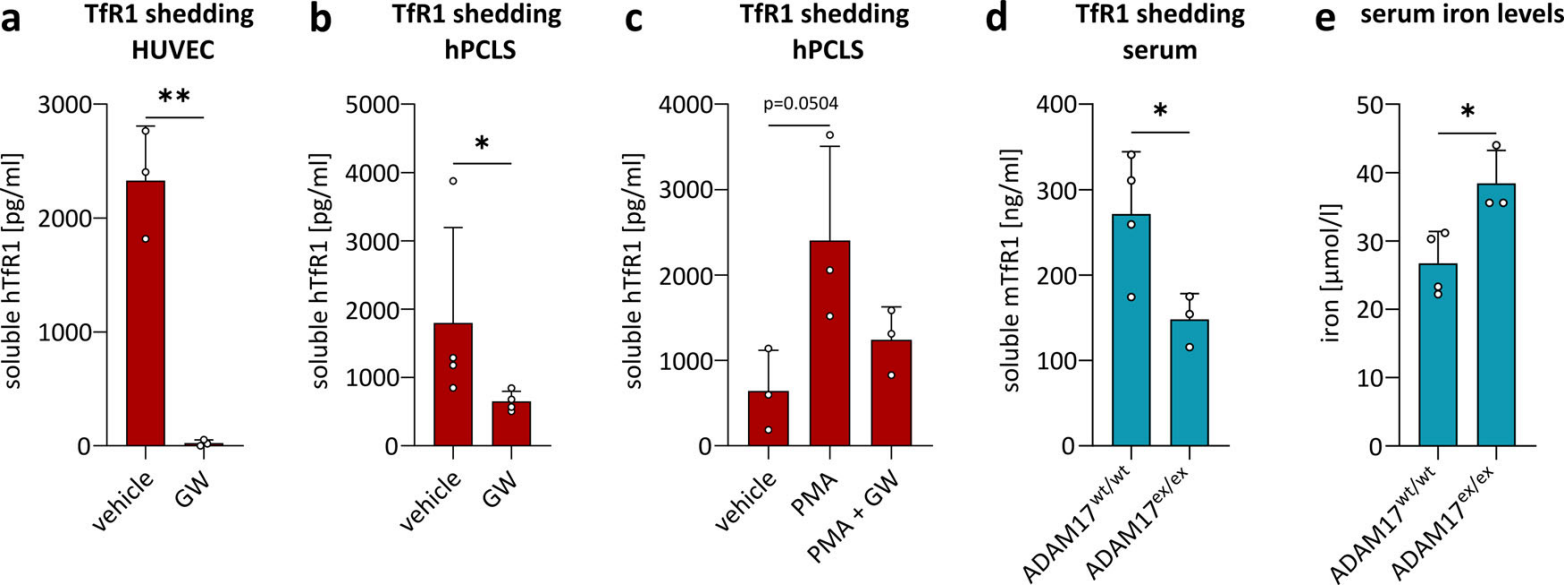
Release of TfR1 ex vivo and in vivo depends on ADAM17/ADAM10 activity. **a**–**c**, ELISA was used to assess soluble TfR1 levels. Cells or lung slices were subjected to the following treatments: HUVECs: 10 µM GW for 24 h (**a**) (*n* = 3); hPCLS: 50 µM GW (**b**) (*n* = 4) and/or 0.5 µM PMA for 24 h (**c**) (*n* = 3). DMSO was used as a vehicle control. **d**,**e**, Soluble TfR1 levels (**d**) (*n* ≥ 3) and iron levels (**e**) (*n* ≥ 3) were quantified in the serum of ADAM17^wt/wt^ and ADAM17^ex/ex^ mice. Data are presented as mean + s.d. from at least three independent experiments. Significant differences are indicated by asterisks (*P < 0.05, **P < 0.01, ***P < 0.001).

To demonstrate the physiological relevance of ADAM10 and ADAM17 for TfR1 shedding, we investigated TfR1 shedding in primary endothelial cells. TfR1 is constitutively shed by HUVECs, which can be inhibited by the ADAM10/ADAM17 inhibitor GW (Fig. 7a) and the ADAM10 inhibitor GI254023X (Supplementary Fig. 7). Furthermore, soluble TfR1 is released from ex vivo human precision-cut lung slices (hPCLS) and can be increased by PMA (Fig. 7b,c). This release is again inhibited by GW (Fig. 7b,c).

In addition, we examined soluble TfR1 levels in the in vivo mouse model of ADAM17-hypomorphic mice, with only around 5% ADAM17 activity left^52^. Indeed, circulating soluble TfR1 levels were reduced in ADAM17^ex/ex^ mice compared with wt mice (ADAM17^wt/wt^). Interestingly, serum iron levels were increased in ADAM17^ex/ex^ mice (Fig. 7d,e).

Overall, ADAM10 and ADAM17 are key sheddases of TfR1 ex vivo and in vivo and hence can influence the systemic iron homeostasis in mice and humans.

## Discussion

Many (patho)physiological processes including cancer suppression, inflammation and infection control that are crucial for the health and survival of the organism are linked to well-regulated ferroptosis. Hence, homeostasis and metabolism of the essential metal iron is tightly regulated. TfR1 is the dominant entry point for iron in most cells. The rate at which Tf binds and is taken up into cells depends on the availability of surface TfR1. This effectively limits how much iron a cell can acquire, making it a regulatory restriction point for the iron supply to the cellular metabolism.

By reanalyzing mRNA sequencing data of iRhom2-deficient patients we showed a connection between iRhom2 and iron metabolism. The loss-of-function mutation in *RHBDF2* results in immunodeficiency^31^ and increased *TFRC* expression. However, the dysregulated iron homeostasis cannot simply be attributed to infection-induced inflammation, as comparison to patients with chronic inflammation^32,33^ revealed distinctly regulated iron-related genes (Fig. 1 and Supplementary Fig. 1). Notably, the dysregulation of TfR1, for example, by a point mutation (TfR1^R22W^, within the endocytosis motif (YTRF)), leading to increased TfR1 levels at the cell surface^34^, even showed a reciprocal regulation with decreased expression of *RHBDF2, ADAM17* and *ADAM10* in peripheral blood mononuclear cells (Supplementary Fig. 1).

The direct connection between TfR1 and iRhom2 was until now unknown. Yet, our interactome analysis^25^ revealed a potential interaction between iRhom2 and TfR1. By coIPs, we confirmed that both iRhom1 and iRhom2 interact with TfR1. We previously identified ADAM17 as a protease for TfR1, and here, we directly demonstrate that iRhoms can bind ADAM17 substrates. Our findings establish iRhoms as a TfR1 substrate platform, promoting ADAM17-mediated shedding. Furthermore, we were able to demonstrate an interaction of the already known ADAM17 substrates TGFα and AREG with iRhom2, showing that the results found here may also apply to other ADAM17 substrates. This further supports the hypothesis of coselectivity of iRhoms for ADAM17 substrates^22,23^. The binding between TfR1 and iRhom2 occurs beyond the ER, possibly in the Golgi. However, this interaction is not limited to the secretory pathway, as we also showed TfR1–iRhom2 binding at the cell surface. The cellular compartment in which iRhoms interact with ADAM17 substrates may vary depending on the substrate. Previous studies have shown that iRhom1 and iRhom2 codetermine the substrate specificity for ADAM17^22,23^. The novel ADAM17 substrate TfR1 exhibits a stronger interaction with the pro-inflammatory iRhom2 than with iRhom1. Given that iRhom1 is expressed nearly ubiquitously, whereas iRhom2 is predominantly found in immune cells^16^, their differential binding to TfR1 might codetermine the localization of its shedding. Notably, iRhom1 and iRhom2 differ most substantialy in their long N-terminal regions^40^, which we identified as essential for TfR1 binding. The N terminus of iRhom has been shown to impact ADAM17 binding, stability and shedding activity. It is regulated through interactions with various partners, including FRMD8^53,54^, 14-3-3 proteins^41,46,47^ and ERK1/2^55^. Moreover, the cytosolic N-terminal tail of iRhom2 contains numerous phosphorylation sides^46,47^. This complex, fine regulation may also influence the substrate binding of iRhoms. We were able to provide evidence that the iRhom substrate binding may be phosphorylation dependent, as a phospho-dead mutant of iRhom2 exhibited reduced TfR1 binding. Future research should investigate whether the iRhom–ADAM17 substrate interaction is direct or mediated by other proteins, such as FRMD8 or 14-3-3, and whether this mechanism applies to other ADAM17 substrates.

By contrast, the TspanC8 tetraspanins (Tspan5, Tspan10, Tspan14, Tspan15, Tspan17 and Tspan33) exert differential control over the maturation, trafficking and substrate selectivity of ADAM10. While the molecular basis of this selectivity remains unclear, a recent structural model suggests that different TspanC8 proteins impose distinct steric conformations on ADAM10, thereby regulating access to substrate cleavage sites^56^. The regulation of ADAM10-dependent TfR1 shedding by different Tspans remains to be addressed in future studies.

The function of the soluble forms of various ADAM17 substrates has already been examined in greater detail; however, the role of soluble TfR1 has not yet been fully clarified. While the main function of membrane-bound, noncleaved TfR1 is to facilitate iron uptake into the cell^5,57^, it also serves as a receptor for viruses and other pathogens^58–61^, as well as for the immunoglobulin IgA^62^. Thus, cleavage of the receptor might reduce viral or pathogenic infection. Although *TFRC* gene expression is tightly regulated by the iron-responsive element and iron regulatory proteins^4^, this represents a long-term regulatory mechanism in contrast to the more immediate effects of endocytosis or surface TfR1 cleavage. Following endocytosis, TfR1 is typically recycled back to the cell surface^63^. Therefore, apart from exosomal release, cleavage may represent the most rapid mechanism for reducing TfR1 surface levels and hence iron uptake.

As tight regulation of TfR1 surface expression is crucial for maintaining iron homeostasis, our results indicate that distinct compensatory responses occur when TfR1 shedding is disrupted. The loss of either ADAM17 or ADAM10 is associated with the downregulation of TfR1 gene expression, which indicates transcriptional compensation. In the absence of ADAM17, increased surface availability of ADAM10 partially compensates for the loss of ADAM17 activity. However, this is insufficient to normalize TfR1 surface levels, resulting in elevated TfR1 abundance and increased sensitivity to ferroptosis.

Functional redundancy and compensation of sheddases for different substrates have generally already been described in literature^64^. Our results also do not rule out the possible involvement of other sheddases alongside ADAM10 and ADAM17. A few proteases have been associated with the release of TfR1. However, conclusive evidence for direct cleavage and its physiological relevance are still limited. Guillemot et al. (2013) and Dion et al. (2022) proposed a role for proprotein convertase 7 (PC7)^65^ and TMPRSS6 (also known as matriptase-2)^66^ in the release of TfR1. Furthermore, Kaup et al. (2002) concluded from inhibition data that members of the ADAM family may shed TfR1^11^. In our study, we now identified ADAM10 and ADAM17 as major physiological TfR1 sheddases. Both proteases mediate constitutive TfR1 shedding, while ADAM17-dependent shedding can be further enhanced for example upon PKC activation by PMA. Interestingly, proprotein convertase 7 is important in ADAM10 and ADAM17 maturation^39,67,68^. This may explain the effects of proprotein convertase 7 in the release of soluble TfR1.

In addition to protease-mediated shedding, TfR1 release via extracellular vesicles has been described^48^. This may explain the PMA-stimulated release of TfR1 observed in HEK293 ADAM10/ADAM17 dKO cells. While this release is independent of ADAM10 and ADAM17 activity, it is known that PMA can induce exosome secretion^50,69^. Notably, PMA-stimulated exosome release is not inhibited by the ADAM10/ADAM17 inhibitor GW, whereas PMA-stimulated ADAM17-mediated TfR1 shedding is susceptible to GW inhibition. In addition to the pharmacological inhibition and CRISPR–Cas9-based KO approaches in a human model cell line, we also demonstrated ADAM10- and ADAM17-dependent TfR1 shedding in ex vivo models, including primary endothelial cells (HUVECs) and hPCLS, as well as in an in vivo ADAM17-hypomorphic mouse model. Moreover, we observed increased total TfR1 protein levels in ADAM10/ADAM17 dKO cells, as well as elevated TfR1 surface levels in ADAM17 KO and ADAM10/ADAM17 dKO cells. With regard to the measurement of soluble TfR1 as a clinical parameter, it has been stated that the surface levels of TfR1 correlate with the amount of soluble TfR1^9,70^. However, our findings at the cellular level demonstrate a more complex regulatory process involving the activity of ADAM10 and ADAM17 that are susceptible to modification under different conditions. Therefore, TfR1 shedding should be examined more closely, particularly in contexts such as inflammation, iron deficiency and iron overload, with special regard to iRhom1/2, ADAM10 and ADAM17 expression and activity.

Excessive iron uptake can result in ferroptosis, an iron-dependent type of cell death^2^. Here, we demonstrated that ADAM10 and ADAM17 KO as well as ADAM10/ADAM17 dKO lead to increased Tf uptake. This is in line with a significant increase of RSL3-inducable ferroptosis in this KO cells. However, the KO cells differ during long-term RSL3-induced ferroptosis. In this context, the presence of ADAM17 exerts a long-term protective effect in ADAM10 KO cells, supporting the hypothesis that ADAM17-dependent TfR1 shedding plays a critical role in limiting excessive iron uptake and preventing ferroptosis. Interestingly ferroptosis is known to inhibit cancer progression^3^. Noteworthy, ADAM17 is already discussed as an important target for promising cancer therapies^71^. As we have shown that targeting ADAM17 also induces ferroptosis, this is another mechanism which could be exploited in anticancer treatments.

In conclusion, we identified iRhoms as a substrate platform for the novel ADAM17 substrate TfR1. It preferentially binds to pro-inflammatory iRhom2 over iRhom1, with the cytosolic N terminus of iRhom serving as the crucial binding determinant. Using CRISPR–Cas9-based KO in a human model cell line, as well as pharmacological inhibition in ex vivo models, we demonstrated that TfR1 is a shared substrate of ADAM10 and ADAM17. Functionally, ADAM17-dependent TfR1 shedding reduces excessive iron uptake and can thereby prevent ferroptosis. Furthermore, data from ADAM17-hypomorphic mice revealed that reduced TfR1 shedding correlates with elevated serum iron levels, highlighting the systemic relevance of ADAM17-mediated TfR1 shedding in patho(physiological) iron homeostasis.

## Supplementary information

Supplementary Information

## Data Availability

Previously published interactome data are available at 10.1007/s00018-021-03845-3 (^25^). The datasets and materials generated during the current study are available from the corresponding author upon reasonable request.
