# Supplementary material for: Transferrin receptor 1 shedding by the pro-inflammatory iRhom–ADAM17 complex and ADAM10 regulates cellular iron uptake and ferroptosis
Source: Exp Mol Med. 2026 Jun 4;58(6):1824–37. doi: 10.1038/s12276-026-01731-1 (PMC13324561; doi:10.1038/s12276-026-01731-1)
Supplement: Supplementary file 1 — Supplementary Information [file 12276_2026_1731_MOESM1_ESM.pdf]

Supplementary methods

Generation of iRhom2 knockout and iRhom1/iRhom2 double knockout HEK293 cells

The GeneArt CRISPR Nuclease CD4 vector (Thermo Fisher Scientific, A21175) was used to generate knockout cell lines. Target sequences were selected using CRISPick (Broad Institute; Human GRCh38/NCBI RefSeq v.108.20200622 as the reference genome), with the following guide RNAs designed: human iRhom1 target 1 – CGCCACCCCCACTCTACGTG; target 2 – GGACATGGACGACCACAGGT; target 3 – AGGTGCCATCCCTAACGGAG; human iRhom2 target 1 – GCTTGCCTTACTAAAAAAGG; target 2 – GGAGTAGCCAGAACGGACACAC; target 3 – AGTGTCATCCTGCCACTTGA. HEK293 cells (4 × 10^6) were transfected using Lipofectamine 3000. After 24 hours, CD4-positive cells were isolated using the Dynabeads™ CD4 Positive Isolation Kit (Thermo Fisher Scientific, 11331D), in accordance with the manufacturer's protocol. Clonal populations were obtained by limiting dilution (one clone per well). To confirm gene knockout, genomic DNA (gDNA) was extracted using the Invisorb® Spin Universal Kit (Invitek, 1050100200), following the manufacturer's instructions. Complementary DNA (cDNA) synthesis was performed using the Platinum Direct PCR Universal Master Mix (Thermo Fisher Scientific, A44647200). PCR amplification was conducted under the following conditions: initial denaturation at 95°C for 2 min; followed by 30 cycles of 95°C for 5 s, 60°C for 10 s, and 72°C for 15 s. The primers used were as follows: iRhom1 target 1: forward – TCCCAAGACCATGTCCCCATCCCAAGGTA; reverse – CCCCTTGCTGAGTCCCTG / GGAACCCACTCATCCTCTTATTAGACCTTG; iRhom1 target 2: forward – CGCGATGCCATAGATGC; reverse – GACAGGAGGTGGTGAGCAC; iRhom1 target 3: forward – CTTTAGCGCTGCCTCCGATGGGGACTCGAA; reverse – CCCTCTGCTCCTTCTCCAG / AAGGCCTCTGTGCCTCTGGGTGAGCTAGGA; iRhom2 target 1: forward – GTCTTCACCCTACCCAACAGA; reverse – GGGCTTGATGATTCCTTCGG; iRhom2 target 2: forward – CCAGGTGAGCTTTAGGGGAT; reverse – CATGGGGAAAGGAGGCAGG: iRhom2 target 3: forward – CTACCCACGTCCCTG; reverse – ACTAGCTCCACTTGCCCTG. PCR products were resolved by agarose gel electrophoresis and bands of the expected size were excised and purified using the GeneJET PCR Purification Kit (Thermo Fisher Scientific, K0702). To assess potential frameshift mutations at the CRISPR-Cas9 target sites, Sanger sequencing was performed. Resulting sequences containing insertions or deletions were analysed using SnapView to determine whether these mutations led to transcription termination within exon regions, thereby producing non-functional proteins. Clonal cell lines exhibiting gene knock-out at the genomic level, as well as supporting evidence from cellular analyses (Supplementary Fig. 2n-p, Supplementary Fig. 5b) were identified as confirmed knockout clones.

HA-tag based co-immunoprecipitation (coIP) from cell lysate

In the precipitation experiments, a total of 4.0 × 10^6 HEK293 cells, which either stably expressed HA-tagged iRhom variants or GFP (negative control, ctr) were lysed in 500 µl of lysis buffer. The lysis buffer consists of 50 mM Tris, 137 mM NaCl, 2 mM EDTA, and 10 mM 1,10-Phenanthroline, adjusted to a pH of 7.5 and supplemented with 1% Triton X-100 (Sigma-Aldrich) and cOmplete™ protease inhibitor cocktail (Sigma-Aldrich). The resulting cell lysates were subsequently clarified through centrifugation at 16,000 × g for 20 min at 4°C. For co-immunoprecipitation (coIP) assays, 450 µl of the cleared lysate was incubated with 10 µl of anti-HA magnetic beads (ThermoFisher) for a minimum duration of 3 h or overnight, followed by six washes with the lysis buffer. After the lysis buffer was removed, the beads were prepared for analysis via western blotting by the addition of 20 µl of reducing loading buffer, consisting of 3% (w/v) SDS, 16% glycerol, 8% 2-mercaptoethanol, 0.01% (w/v) bromophenol blue, and 0.1 M Tris HCl at pH 6.8, and were subsequently heated at 60°C for 20 min.

HA-tag based coIP of surface proteins

For precipitation of surface proteins, 16 x 10^6 HEK293 cells, stably expressing wt murine iRhom1, wt murine iRhom2 with HA tag or GFP as a negative control, were incubated with anti-HA antibody (1:500, TA150086, ThermoFisher) in PBS for 30 min on ice. After washing the samples twice with PBS, cells were lysed with 1.5 ml lysis buffer. The cell lysates were cleared by centrifugation at 16,000 × g for 20 min at 4°C. Protein A/G magnetic beads (ThermoFisher) were used to precipitate anti-HA antibody that has bound to HA-tagged wt murine iRhom1 or wt murine iRhom2 which were present on the cell surface. The beads were incubated with the lysate for 1 h and afterwards washed for six times with lysis buffer. Following the samples were prepared for western blot analysis as described before.

myc-tag based soluble TfR1 precipitation from supernatant

To precipitate soluble TfR1 from the cell supernatant, the samples were prepared as followed: The medium was exchanged when wt HEK293 cells or wt HEK293 cells stably expressing wt murine iRhom2 with HA tag and TfR1 with myc tag reached a confluence of 80%. The cells were incubated for 4 h with DMEM10% and different treatments (100 nM PMA (CaymanChemical) and/or 10 µM GW280264X (Cayman Chemical)). Supernatants were harvested, supplemented with cOmplete™ protease inhibitor cocktail and cleared from cell debris by centrifugation. Cells were lysed and used as a control for western blot analysis. Anti-myc magnetic beads (ThermoFisher) (20 µl per sample) were used to precipitate soluble TfR1 with myc tag from the supernatant. The beads were incubated with the supernatant for 3 h and subsequently washed six times with lysis buffer. Following the samples were prepared for western blot analysis as described before.

isolation of extracellular vesicles

In order to isolate extracellular vesicles from supernatant, cells were treated like described for *´myc-tag based soluble TfR1 precipitation from supernatant´* for 24 h. Afterwards supernatants were harvested and supplemented with cOmplete™ protease inhibitor cocktail. Cells were lysed and used as a control for western blot analysis. Every following step was carried out at 4°C. Following two centrifugation steps (10 min, 300 × g and 20 min, 2000 x g) the supernatant was transferred into ultracentrifugation tubes (355631, Beckman Coulter) and centrifuged for 30 min at 10,000 x g using the ultracentrifuge (Optima XPN-80, Beckman Coulter). The supernatant was filtered through a filter (pore size: 0.25 µm), transferred into new ultracentrifugation tubes and centrifuged 75 min at 100,000 x g. Afterwards the supernatant was used for a myc-tag based soluble TfR1 precipitation as described in the previous section. The remaining extracellular vesicles within the ultracentrifugation tubes were washed with PBS and centrifuged again for 75 min at 100,000 x g. The PBS was discarded and the extracellular vesicle samples were prepared for western blot analysis as described before.

protein A/G bead based coIP of endogenous TfR1

In order to precipitate endogenous TfR1, 10 µl of magnetic protein A/G beads (ThermoFisher) per sample were preincubated with 1.25 µg anti-TfR1 antibody (13-6800, ThermoFisher) for at least two hours. Lysates of 16 x 10^6 HEK293 cells stably expressing wt murine iRhom2 or GFP as a negative control were incubated with preincubated protein A/G beads for at least 3 h. Beads without previous antibody incubation were used as a negative control. After washing six times with lysis buffer, samples were prepared for western blot analysis as described before.

Supplementary figures

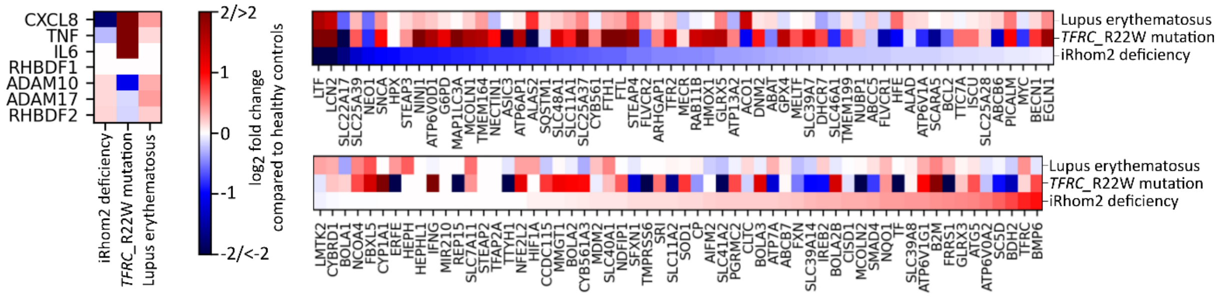

Supplementary Figure 1

Whole blood mRNA sequencing data from patients with congenital iRhom2 deficiency ([26], GSE184876) and those with chronic inflammatory lupus erythematosus ([41,42], GSE112087), and peripheral blood mononuclear cell (PBMC) mRNA sequencing data from a patient carrying a point mutation in TfR1 ($TfR1^{R22W}$) ([43], GSE243237) were reanalyzed. Gene expression profiles from these patient cohorts were compared to those of healthy controls. Changes in gene expression relative to the healthy control group, expressed as $\log_2$ fold change are shown for *IL6, CXCL8 (IL8), TNF, ADAM10, ADAM17, RHBDF1,* and *RHBDF2*. Further, genes associated with the following Gene Ontology (GO) terms[68,69]: GO:0006826 (iron ion transport), GO:0010039 (response to iron ion), GO:0006879 (intracellular iron ion homeostasis) and GO:0097707 (ferroptosis) were compared for the selected data sets.

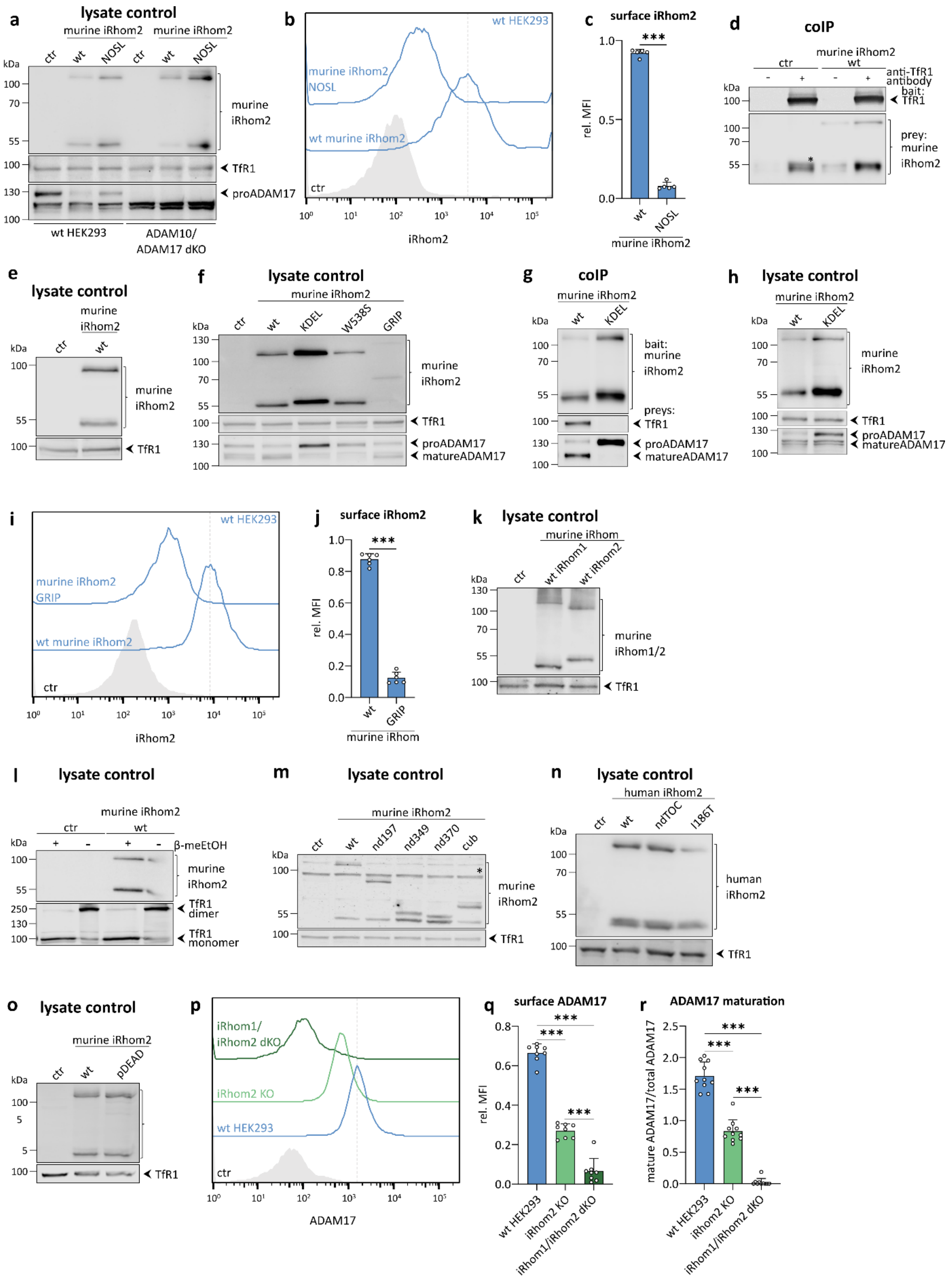

Supplementary Figure 2

Corresponding lysate controls to coIPs of **Figure 2**. HEK293 cells were transduced either with the indicated iRhom variants or GFP (ctr) as a control. Unless stated otherwise, wt HEK293 cells were used. **(a)** lysate control for Figure 2b; additionally, ADAM10/ADAM17 dKO cells were used (*n*=4). **(b), (c), (i), (j)** Cell surface levels of iRhom2 variants with HA tag were measured by flow cytometry and the median fluorescence intensity (MFI) was used for quantification. **(d)** Protein A/G beads, pre-incubated with or

without an anti-TfR1 antibody, were used to precipitate endogenous TfR1 as a bait and co-precipitate wt murine iRhom2 (prey) from cell lysates. The band marked by an asterisk indicates a fragment of the anti-TfR1 antibody used for immunoprecipitation. **(e)** Corresponding lysate control to protein A/G coIP in (d). **(f)** lysate control for Figure 2d. **(g)** Western blot analysis of a coIP using HEK293 cells expressing wt murine iRhom2 or murine iRhom2_KDEL, which was quantified in Figure 2e. The iRhom variants with an HA tag were used as a bait. In **(h)** the corresponding lysate control for (g) is shown (n=3). **(k), (l), (m), (n)** lysate controls for Figures 2f, 2h, 2i, 2k, respectively. In lysate control **(o)** iRhom1/iRhom2 dKO cells were used. **(p)**, **(q)** Cell surface levels of endogenous ADAM17 in wt HEK293, iRhom2 KO and iRhom1/iRhom2 dKO cells were measured by flow cytometry and the median fluorescence intensity (MFI) was used for quantification (*n*=8). ADAM17 surface levels were compared to validate the effect of the iRhom knockouts. **(r)** ADAM17 maturation was determined for wt HEK293, iRhom2 KO and iRhom1/iRhom2 dKO cells by quantifying the ratio of mature ADAM17 per total ADAM17 protein levels (*n*=10). The corresponding representative western blot is shown in Supplementary Fig. 5b. ADAM17 maturation was compared to validate the effect of the iRhom knockouts. (a) *n*=4; (b,c) *n*=5; (d,e) *n*=4; (f) *n*≥3; (i,j) *n*=6; (k) *n*=5; (l) *n*=3; (m) *n*=3, the bands marked with an asterisk indicate unspecific antibody bands; (n) *n*=4; (o) *n*=3. The higher iRhom band represents the full length iRhom and the lower iRhom band represents a cleaved fragment of iRhom[41]. Data are presented as mean + SD from at least three independent experiments. Significant differences were indicated by asterisks (* $p < 0.05$, ** $p < 0.01$, *** $p < 0.001$).

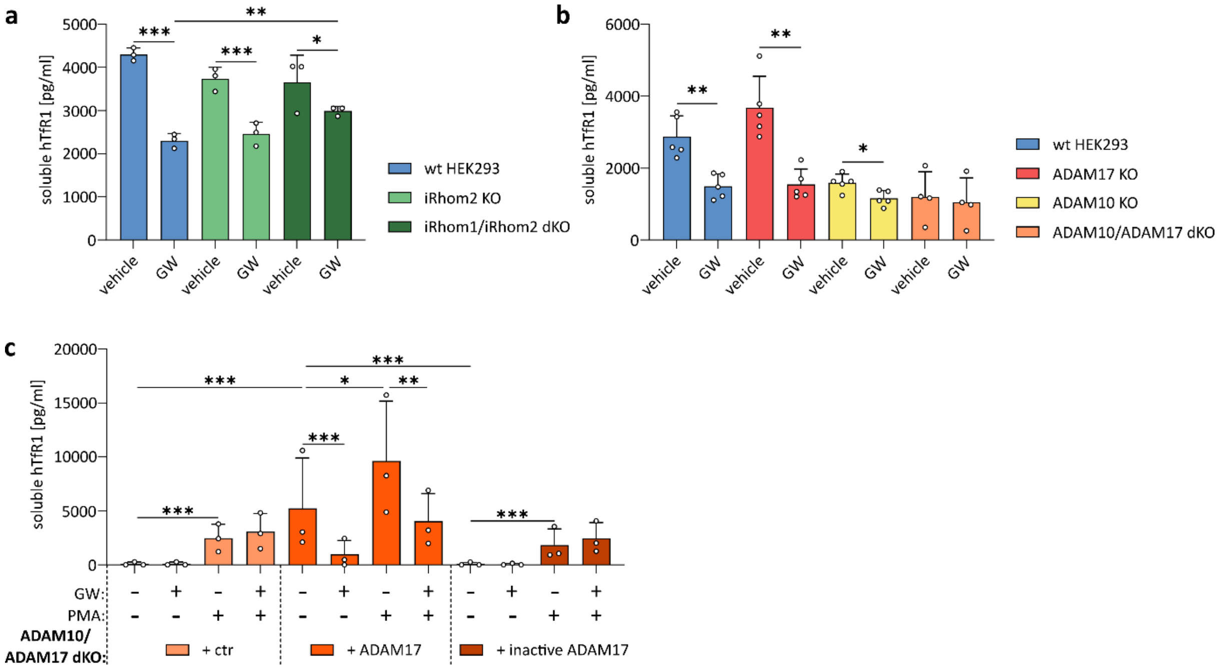

Supplementary Figure 3

Corresponding raw data to **Figure 3.** ELISA was used to determine the amount of soluble human TfR1. iRhom2 KO, iRhom1/iRhom2 dKO and wt HEK293 cells (n =3) **(a)** and ADAM17 KO, ADAM10 KO, ADAM10/ADAM17 dKO and wt HEK293 cells (*n*≥4) **(b)** were utilized. **(a,b)** Cells were incubated for 24 h with 3 µM GW (ADAM10 and ADAM17 inhibitor). DMSO was used as a vehicle control to show constitutive TfR1 shedding. **(c)** ADAM10/ADAM17 dKO cells were transiently transfected with GFP (ctr) as a control, ADAM17 or inactive ADAM17. Cells were incubated for 24 h with 3 µM GW and/or 100 nM PMA (ADAM17 activator) (*n*=3). DMSO was used as a vehicle control (-/-). Data are presented as mean + SD from at least three independent experiments. Significant differences were indicated by asterisks (* p < 0.05, ** p < 0.01, *** p < 0.001).

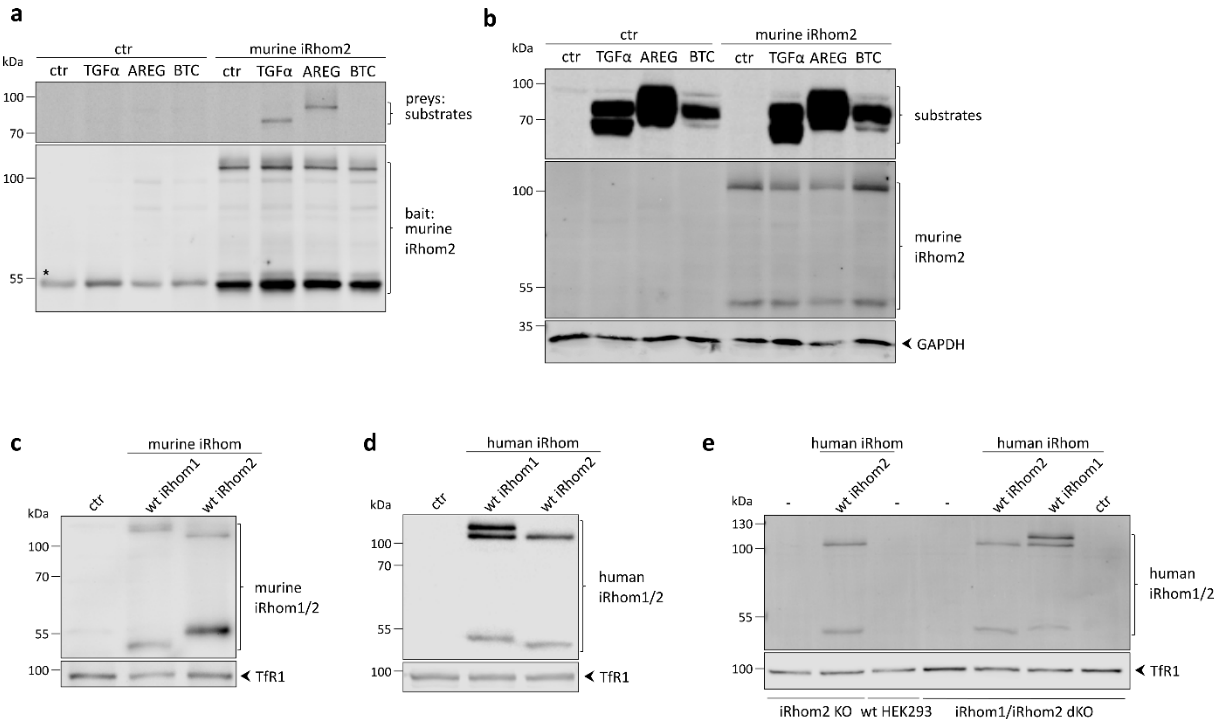

Supplementary Figure 4

(a,b) ADAM17 KO cells were transduced with wt murine iRhom2 or GFP as a control (ctr). Additionally, cells were transfected with alkaline phosphatase (AP) coupled ADAM17 substrates transforming growth factor alpha (TGFα), amphiregulin (AREG), the ADAM10 substrate betacellulin (BTC) or GFP as a ctr. **(a)** HA-tagged iRhom was used as a bait to precipitated AP-coupled substrates as a prey (*n*=3). The asterisk indicates a fragment of the antibody used for immunoprecipitation. Corresponding lysate control is shown in **(b)**. The higher iRhom band represents the full length iRhom and the lower iRhom band represents a cleaved fragment of iRhom[41]. **(c-e)** Lysate controls corresponding to coIPs shown in Figure 4. HEK293 cells stably expressing the indicated iRhom variants or GFP (ctr) as a negative control were used. In **(c)** (*n*=3) and **(d)** (*n*=4) wt HEK293 cells and in **(e)** (*n*=4) wt HEK293 cells, iRhom2 KO cells and iRhom1/iRhom2 dKO cells were used.

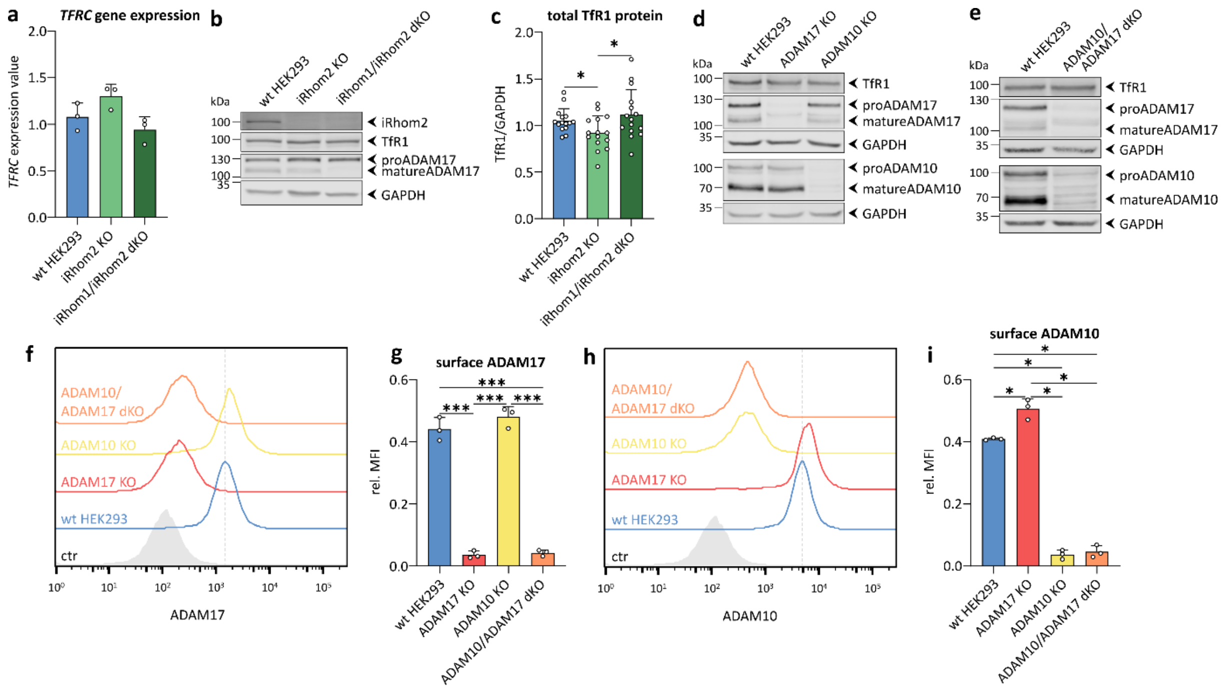

Supplementary Figure 5

(a) The *TFCR* gene expression of wt HEK293, iRhom2 KO and iRhom1/iRhom2 dKO cells was measured by qPCR (*n*=3). **(b)** Lysates of wt HEK293, iRhom2 KO and iRhom1/iRhom2 dKO cells were used to validate the iRhom2 knockout. Unfortunately, there is no reliable antibody available for iRhom1. Nevertheless, the iRhom1/iRhom2 dKO can be indirectly validated by the decrease of mature ADAM17, which is even stronger than the reduction of mature ADAM17 in the iRhom2 KO cells (Supplementary Fig. 2p). **(c)** Western blot of lysates from wt HEK293, iRhom2 KO and iRhom1/iRhom2 dKO cells (Fig. 2b) was used to quantify TfR1 protein levels relative to the loading control GAPDH (*n*=15). **(d)** The same approach was adopted for the western blot analysis of lysates from wt HEK293, ADAM17 KO, ADAM10 KO (*n*=3) and **(e)** ADAM10/ADAM17 dKO HEK293 cells (*n*=3), corresponding to the quantification shown in Figure 5g,h. Flow cytometry was utilized to detect ADAM17 **(f,g)** and ADAM10 **(h,i)** at the cell surface of wt HEK293 cells, ADAM17 KO, ADAM10 KO and ADAM10/ADAM17 dKO HEK293 cells. The median fluorescence intensity was used for quantification **(g,i)** (*n*=3). Data are presented as mean + SD from at least three independent experiments. Significant differences were indicated by asterisks (* $p < 0.05$, ** $p < 0.01$, *** $p < 0.001$).

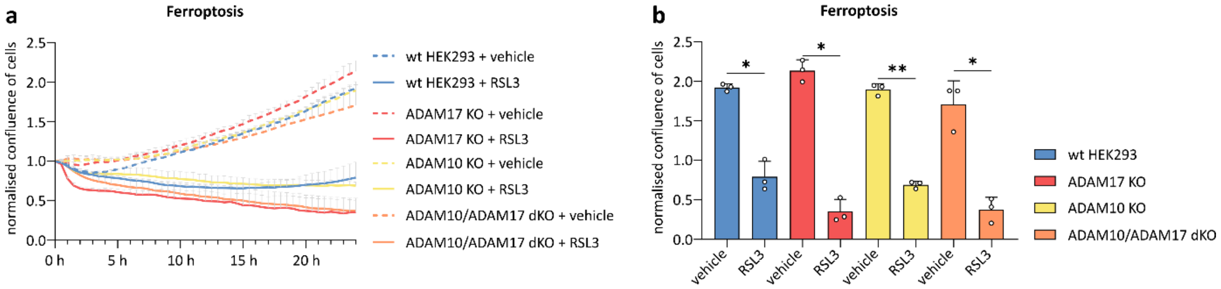

Supplementary Figure 6

Ferroptosis was induced by treating wt HEK293, ADAM17 KO, ADAM10 KO or ADAM10/ADAM17 dKO cells with 10 µM RSL3 for a period of 24 h. DMSO was used as a vehicle control. Confluence was normalized to 0 h (set at 1) and the results are shown for the vehicle control group (dashed lines) and the RSL3-treated group (solid lines) over 24 h **(a)** and at 24 h **(b)** (*n*=3). Data are presented as mean + SD from at least three independent experiments. Significant differences were indicated by asterisks (* $p < 0.05$, ** $p < 0.01$, *** $p < 0.001$).

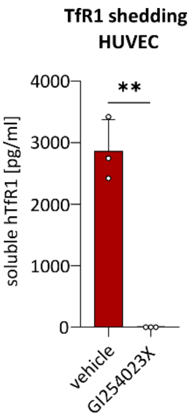

Supplementary Figure 7
Human umbilical vein endothelial cells (HUVECs) were treated for 24 h with the preferential ADAM10 Inhibitor GI254023X (n=3). ELISA was utilized to assess soluble TfR1 levels. DMSO was used as a vehicle control. Data are presented as mean + SD from three independent experiments. Significant differences were indicated by asterisks (* $p < 0.05$, ** $p < 0.01$, *** $p < 0.001$).
